# Early embryo mortality in natural human reproduction: What the data say

**DOI:** 10.12688/f1000research.8937.2

**Published:** 2017-06-07

**Authors:** Gavin E. Jarvis

**Affiliations:** 1 Department of Physiology, Development and Neuroscience, University of Cambridge, Cambridge, CB2 3EG, UK

**Keywords:** early pregnancy loss, occult pregnancy, embryo mortality, human chorionic gonadotrophin, Hertig, pre-implantation embryo loss

## Abstract

How many human embryos die between fertilisation and birth under natural conditions? It is widely accepted that natural human embryo mortality is high, particularly during the first weeks after fertilisation, with total prenatal losses of 70% and higher frequently claimed. However, the first external sign of pregnancy occurs two weeks after fertilisation with a missed menstrual period, and establishing the fate of embryos before this is challenging. Calculations are additionally hampered by a lack of data on the efficiency of fertilisation under natural conditions. Four distinct sources are used to justify quantitative claims regarding embryo loss: (i) a hypothesis published by Roberts & Lowe in *The* *Lancet*  is widely cited but has no practical quantitative value; (ii) life table analyses give consistent assessments of clinical pregnancy loss, but cannot illuminate losses at earlier stages of development; (iii) studies that measure human chorionic gonadotrophin (hCG) reveal losses in the second week of development and beyond, but not before; and (iv) the classic studies of Hertig and Rock offer the only direct insight into the fate of human embryos from fertilisation under natural conditions. Re-examination of Hertig’s data demonstrates that his estimates for fertilisation rate and early embryo loss are highly imprecise and casts doubt on the validity of his numerical analysis. A recent re-analysis of hCG study data concluded that approximately 40-60% of embryos may be lost between fertilisation and birth, although this will vary substantially between individual women. In conclusion, natural human embryo mortality is lower than often claimed and widely accepted. Estimates for total prenatal mortality of 70% or higher are exaggerated and not supported by the available data.

## Introduction

Early human embryo mortality is a matter of considerable interest not only to reproductive biologists and fertility doctors, but also to philosophers ^1, 2^, theologians ^3^ and lawyers ^4^. Most especially, becoming pregnant and having children is of overwhelming and personal importance to many women and their families. As with all biological processes, nothing works perfectly all the time ^5^, and failure to conceive and pregnancy loss are common problems. However, among reputable scientific publications, including medical and reproductive biology text books, scientific reviews and primary research articles, reported mortality estimates are surprisingly varied and include: 30–70% ^6^, >50% ^7^ and 75% ^8^ before and during implantation; >50% ^9^, 73% ^10^ and 80% ^11^ before the 6 ^th^ week; 75% before the 8 ^th^ week ^12^; 70% in the first trimester ^13^; 40–50% in the first 20 weeks ^14^; and 46% ^7^, 49% ^15^, 50% ^16– 18^, >50% ^19, 20^, 53% ^21^, 54% ^22^, 60% ^23^, >60% ^24^, 63% ^25, 26^, 70% ^27– 31^, 50–75% ^32^, 76% ^10, 33^, 78% ^34^, 80–85% ^35^, >85% ^36^, and 90% ^37^ total loss from fertilisation to term. The variance in these estimates is striking and the scale of some implausible. 90% intrauterine mortality implies a maximal live birth fecundability of 10%, and only then if all other stages of the reproductive process are 100% efficient. Observed human fecundability is low compared to other animals ^21^, but at approximately 20–30% ^9, 38^ it is still higher than implied by such a high embryo mortality rate. Such inconsistent estimates of pregnancy loss are not reassuring, nor do they provide a sound basis for either a quantitative understanding of natural human reproductive biology or an unbiased appraisal of artificial reproductive technologies. These divergent and excessive values therefore invite scrutiny of the evidence that supports them. In this article, I identify and re-evaluate published data that contribute to claims regarding natural human embryo mortality. Using the available data, I attempt to answer the question: “ *How many human embryos die between fertilisation and birth under natural conditions?*”

## A quantitative framework for embryo mortality

A quantitative framework has recently been proposed to facilitate the calculation and comparison of embryo mortalities from fecundability and pregnancy loss data ^39^. Briefly, the model comprises conditional probabilities ( *π*) of the following biological processes: (1) reproductive behaviours resulting in sperm-ovum-co-localisation per cycle = *π _SOC_*; (2) successful fertilisation given sperm-ovum-co-localisation = *π _FERT_*; (3) implantation of a fertilised ovum as indicated by increased levels of human chorionic gonadotrophin (hCG) = *π _HCG_*; (4) progression of an implanted embryo to a clinically recognised pregnancy = *π _CLIN_*; (5) survival of a clinical pregnancy to live birth = *π _LB_* .

Fecundability ( *FEC*) is the probability of reproductive success per cycle, but may take different values depending on the definition of success. The following four fecundabilities broadly follow Leridon (1977) ^38^:

**1.** Total (all fertilisations): *FEC _TOT_* = *π _SOC_ × π _FERT_*

**2.** Detectable (implantation): *FEC _HCG_* = *π _SOC_ × π _FERT_ × π _HCG_*

**3.** Apparent (clinical): *FEC _CLIN_* = *π _SOC_ × π _FERT_ × π _HCG_ × π _CLIN_*

**4.** Effective (live birth): *FEC _LB_* = *π _SOC_ × π _FERT_ × π _HCG_ × π _CLIN_ × π _LB_*

Hence, the probability that a fertilised egg will perish prior to implantation is [1 - *π _HCG_*], and prior to clinical recognition is [1 - ( *π _HCG_ × π _CLIN_*)]. In theory, embryonic mortality may be estimated at different stages; however, in practice, this depends on available data. Clinical and live birth fecundabilities are most easily quantified and most frequently reported. Total and detectable fecundabilities are less frequently reported, although of direct relevance.

**Figure 1.**
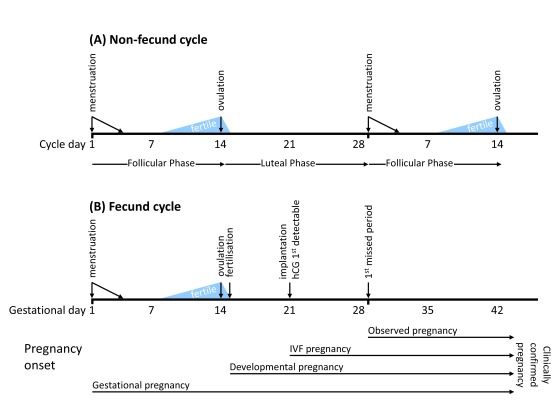
Schematic representation of timelines and key events in ( **A**) non-fecund and ( **B**) fecund menstrual cycles. Menstrual cycle lengths vary considerably and most fall within a range of 20 to 40 days ^45^. A typical menstrual cycle is usually represented as lasting for 28 days, as shown here. Differences in cycle length are mostly due to variations in the duration of the follicular phase, the time from the onset of menstruation to ovulation. The time from ovulation to the onset of the next cycle, the luteal phase, is more consistently 14 days. Therefore, in the typical 28 day cycle, ovulation occurs midway at around 14 days. The fertile period (shown in light blue) is the time during which coitus may result in a pregnancy. The probability of pregnancy is highest when coitus occurs in the two days leading up to ovulation ^40^. In a normal fecund cycle, fertilisation occurs within hours of ovulation in the fallopian tube, after which point an embryo is present and development begins. Embryonic development may fail at any stage from fertilisation through to birth. 6–7 days after fertilisation, the embryo begins to implant in the uterine wall at which stage human chorionic gonadotrophin (hCG) produced by the embryo becomes detectable in urine or serum samples. The onset and duration of pregnancy may be defined in various ways: gestational pregnancy (typically used in clinical practice) is timed from the first day of the last menstrual period; developmental pregnancy begins with fertilisation; in an IVF treatment cycle, although an embryo is present in the uterus immediately following embryo transfer, pregnancy is not considered to be established until there is evidence of implantation, usually provided by elevated hCG levels. The earliest point at which a woman could observe that she is pregnant is approximately 14 days after ovulation/fertilisation with the first missed period. The stage at which pregnancies are clinically confirmed depends on study design and clinical practice, and may be at gestational day 28 (i.e., first missed menstrual period, Zinaman (1996) ^46^, French & Bierman (1962) ^47^), gestational day 42 (Wang (2003) ^48^), or following a positive pregnancy test (Wilcox (1988) ^49^) or satisfactory ultrasound scan.

To aid understanding of the reproductive processes described in this article definitions of key terms have been provided in Box 1, and Figure 1 illustrates the timelines for key biological events associated with fecund and non-fecund cycles.

BOX 1: **Glossary of Key Reproductive Terms**

1. **Ovum**: A female gamete, also known as an egg or oocyte. Ova ( *pl*) are produced by the ovaries of the woman.
2. **Spermatozoon**: A male gamete. Sperm (or spermatozoa, *pl*) are produced in the testes of the man.
3. **Ovulation**: The release of an ovum from the ovary. In humans, ovulation usually involves the release of a single egg in each menstrual cycle.
4. **Fallopian tube**: A narrow tubular extension of the uterus, which opens out next to the ovary. It is also called the oviduct. Following ovulation, the ovum passes into the opening of the fallopian tube and travels towards the uterus.
5. **Coitus**: An act of sexual intercourse between a man and woman, usually resulting in the deposition of sperm within the reproductive tract of the woman.
6. **Menstrual cycle**: An interval of approximately 28 days, which commences with the onset of menstruation. Ovulation occurs mid-way though a menstrual cycle, approximately 14 days before the onset of the next cycle.
7. **Amenorrhoea**: The absence of menstruation. A missed menstrual period is often the first observable sign that pregnancy has commenced, although there are many other causes.
8. **Fertile period**: The time in a woman’s menstrual cycle during which coitus may result in pregnancy. This period probably varies considerably between women. Coitus up to 6 days prior to and 1 day after ovulation may result in pregnancy although the most fertile days are the day of ovulation and the 2 days beforehand ^40^.
9. **Fertilization**: The fusion of a spermatozoon and an ovum, which usually takes place in the fallopian tube up to 24 hours after ovulation.
10. **Conception**: A biologically imprecise term meaning either ‘the coming into existence of a new human being’ or ‘the beginning of a pregnancy’. It is often used synonymously with fertilisation but may also refer to implantation.
11. **Embryo**: A newly fertilised ovum until the eighth week of development.
12. **Zygote**: The newly fertilised ovum: a one-cell embryo.
13. **Blastocyst**: An embryo approximately 5–6 days after fertilisation.
14. **Implantation**: The biological process that begins when a blastocyst attaches to the lining of the uterus approximately 6–7 days after fertilisation. The embryo subsequently becomes embedded within the uterine lining.
15. **Human chorionic gonadotrophin** (hCG): A protein produced by the embryo. It signals to the mother that an embryo is present and prevents menstruation and the loss of the embryo. Elevated levels of hCG can be detected in the serum or urine of a woman from around the time of implantation.
16. **Fecundability**: A measure of reproductive potential. It is the probability of becoming pregnant in a single menstrual cycle. **Fecundity** is often used to mean the probability of achieving a live birth in a single cycle. A **fecund** cycle is one in which fertilisation occurs.
17. **Pregnancy**: The condition of a woman harbouring an embryo, fetus or unborn child. When pregnancy begins is a matter of some confusion ^7^ ( Figure 1). Pregnancy may be considered to commence with fertilisation and lasts approximately 38 weeks. Clinicians often time the onset of pregnancy from day 1 of the last menstrual cycle, 2 weeks before fertilisation, and refer to subsequent time as a period of **gestation**. On this account, pregnancy or gestation lasts approximately 40 weeks. Some scientists and legal judgements define pregnancy as beginning with implantation, one week after fertilisation. This definition is of particular utility in the context of IVF treatment where evidence of implantation is the earliest sign that a transferred embryo has developed normally and that fertility treatment has, up to that point, been successful. For some women, the start of a pregnancy may be noted with the first missed menstrual period, approximately 2 weeks after fertilisation, or a positive pregnancy test.
18. **Miscarriage**: The premature termination of a pregnancy leading to loss of a developing embryo or fetus. Embryo loss may occur before a woman knows she is pregnant. Miscarriage late in pregnancy is often called abortion, with a cut-off of approximately 20 weeks gestation used to distinguish between miscarriage and abortion.
19. **Early Pregnancy Loss**: This usually refers to the loss of an embryo very early in pregnancy, even before a clinical diagnosis is made, when a woman would not be aware of the pregnancy. Such losses are also called **occult**, because they are hidden, or **biochemical,** because they can only be identified by detecting hCG. Pregnancy loss shortly after a clinical diagnosis may also be described as **early**.

## What the data say

Publications containing data relevant to early human embryo mortality were identified primarily by manually tracing citations found in articles, reviews and textbooks. A PubMed search ("early pregnancy loss" [All Fields]) identified some, but not all relevant studies. Certain studies were not conducted to address the specific question, and others are in books or publications that are not adequately indexed. If not entirely complete, nevertheless the data presented form a substantial proportion of relevant, available scientific data on natural early human embryo mortality.

Studies that contribute analysis and data relevant to the quantification of natural human embryo mortality fall into the following four categories and will be considered in turn.

**1.** A speculative hypothesis published in *The Lancet*.

**2.** Life tables of intra-uterine mortality.

**3.** Studies of early pregnancy by biochemical detection of hCG.

**4.** Anatomical studies of Dr Arthur Hertig and Dr John Rock.

### 1. Where have all the conceptions gone?

In 1975, a short hypothesis published in *The Lancet* entitled “ *Where Have All The Conceptions Gone?*” concluded that 78% of all conceptions were lost before birth ^34^. It has been widely cited by both scientists ^9, 25, 27, 28, 41^ and non-scientists ^42, 43^ alike. Conceptions among married women aged 20–29 in England and Wales in 1971 were estimated and compared to infants born in the same period. In this analysis ( Table 1) there are reliable values, e.g., census data, and simple arithmetical calculations. However, speculative values are necessary to perform the calculations. Three are biological: (1) fertilisation rate following unprotected coitus during the fertile period was estimated as 50% and supported by reference to Hertig ^44^ (although his estimate was 84% ^5^); (2) the length of a menstrual cycle (28 days); and (3) the duration of the fertile period (2 days). These latter values are plausible, but also variable ( Figure 1). No justification is provided for three behavioural variables: (1) coital frequency estimated at twice per week; (2) proportion of unprotected coital acts estimated at 25%; and (3) either a random or regular distribution of coital acts during menstrual cycles such that 1/14 of all coital acts fall within a fertile period.

The validity of Roberts & Lowe’s conclusion depends largely on the accuracy and precision of these speculative values. The following two simple analyses illustrate the sensitivity of their conclusion on the speculative values.

**1.** When four of the speculative values are reduced by 25% (e.g., coital frequency reduced to 1.5/week) and cycle length increased by 10% (from 28 days to 31 days ^38^), the estimate for embryo loss drops to 22%. The opposite operation (e.g., coital frequency increased to 2.5/week) results in an estimate of 92% ( Table 1). Embryo loss of 22% is barely sufficient to account for observed clinical losses, and 92% indicates a maximum *FEC _LB_* of 8%. Neither scenario is biologically plausible.

**Figure 2.**
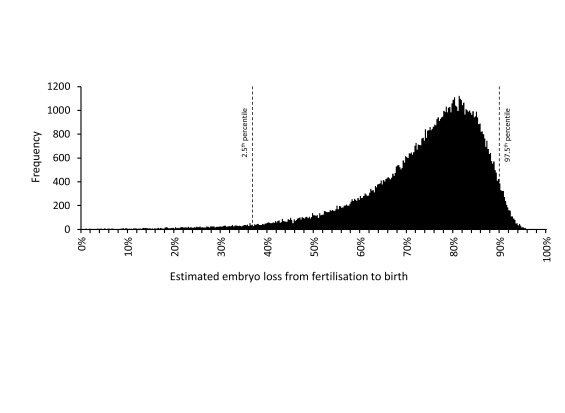
Distribution of embryo loss estimates from fertilisation to birth derived using a modified version of the model of Roberts & Lowe ^34^. Embryo loss values were calculated using alternative speculative values (see text and Table 1) obtained by randomly sampling from normal distributions with means equal to the Roberts & Lowe values and a coefficient of variation of 20%. 100,000 simulated embryo loss values were obtained. Frequencies within a bin size of 0.25% are shown. The 2.5 ^th^ and 97.5 ^th^ percentiles are indicated. The simulation was performed using NONMEM 7.3.0® (Icon PLC, Dublin, Eire). Simulated values are in Dataset 1.

**2.** A non-zero variance was applied to each speculative value reflecting their uncertain nature. Using the random number generator in Microsoft ^®^ Excel (Office 2010) simulated values were obtained by random sampling from normal distributions with means equal to Roberts & Lowe’s speculative values with coefficients of variation equal to 20%. For simplicity, it was assumed that there was no covariance between the different speculative values. Table 1 shows the expected range within which 95% of these simulated values fall (e.g., coital frequency is 1.2–2.8/week). For each simulated record, a new estimate of embryo loss was calculated, and from 10,000 of these, the mean, median and 2.5 ^th^ and 97.5 ^th^ percentiles of embryo loss were determined. This step was repeated 1,000 times: the mean value of the simulated means was 73.3% and of the simulated medians was 76.5%. The mean values of the 2.5 ^th^ and 97.5 ^th^ percentile boundaries for embryo loss were 37% and 90% ( Table 1). Separately, the same simulation was performed using NONMEM 7.3.0 ^®^ (Icon PLC, Dublin, Eire) to generate 100,000 data records which are represented in Figure 2. The code and simulated data values are in Dataset 1.

**Table 1.** Numerical estimates of conceptions and their loss in married women aged 20–29 in England and Wales in 1971. The table replicates the values and calculations of Roberts & Lowe ^34^ with more explanatory detail. In addition, it illustrates how introducing variance into speculative estimates influences the final calculated value of embryo loss. ^*^Data type indicates whether the numerical value is reliable (e.g., derived from census data), the result of a simple arithmetical calculation, or speculative (shown in *italics*). ^§^Values are the 2.5 ^th^ and 97.5 ^th^ percentile boundaries, assuming a normal distribution for the variables centred on Roberts & Lowe’s values with a coefficient of variation of 20%. ^†^Speculative values were adjusted either up or down by 25% compared to Roberts & Lowe’s values. Values for ‘Length of menstrual cycle’ were adjusted by 10%. ^‡^The mean values of the 2.5 ^th^ and 97.5 ^th^ percentile boundaries from 1,000 simulations, each containing 10,000 separate estimates for embryo loss. The derivation of these values is described in the text. Briefly, each separate estimate of embryo loss was calculated using variable speculative values that were obtained by random sampling from a normal distribution with a mean equal to the Roberts & Lowe value and a coefficient of variation of 20%. The mean value of the mean percentage loss was 73.3% and of the median was 76.5%. ^¥^The most frequent duration of a menstrual cycle is 28 days but there is substantial variability and the mean length is generally 30–31 days ^38^.

Description of data	Data type*	Roberts & Lowe values	Low estimate values ^†^	High estimate values ^†^	95% data range (CV = 20%) ^§^
Married women aged 20–29 in 1971	Reliable value	2,437,000	2,437,000	2,437,000	-
*Frequency of coitus per* *married woman per week*	*Speculative* *value*	*2*	*1.5*	*2.5*	*[1.2, 2.8]*
Weeks per year	Reliable value	52	52	52	-
Acts of coitus among married women per year	Calculation	253,448,000	190,086,000	316,810,000	-
*Percentage of acts of coitus* *that are unprotected*	*Speculative* *value*	*25%*	*19%*	*31%*	*[15%, 35%]*
Acts of unprotected coitus per year	Calculation	63,362,000	35,641,125	99,003,125	-
*Length of menstrual cycle* *(days)*	*Speculative* *value*	*28*	*31* ^¥^	*25*	*[17, 39]*
*Length of fertile period in* *each cycle (days)*	*Speculative* *value*	*2*	*1.5*	*2.5*	*[1.2, 2.8]*
Acts of unprotected coitus during fertile period per year	Calculation	4,525,857	1,735,769	9,821,739	
*Probability of fertilisation*	*Speculative* *value*	*50%*	*38%*	*63%*	*[30%, 70%]*
Total fertilised ova per year	Calculation	2,262,929	650,913	6,138,587	-
Number of infants born (live and still) in 1971	Reliable value	505,000	505,000	505,000	-
Total number of lost embryos in 1971	Calculation	1,757,929	145,913	5,633,587	-
**Percentage of embryos** **lost before live birth**	**Calculation**	**78%**	**22%**	**92%**	**[37%, 90%] ^‡^**

Figure 2 data See README.docx for a description of the file. Click here for additional data file. Copyright: © 2017 Jarvis GE 2017 Data associated with the article are available under the terms of the Creative Commons Zero "No rights reserved" data waiver (CC0 1.0 Public domain dedication).

The sole purpose of these simple sensitivity analyses is to illustrate that modest adjustments to Roberts & Lowe’s original speculative values can result in any biologically plausible estimate for embryo loss. Whilst their analysis is useful for highlighting factors that influence observed fecundity, the output from the calculation remains substantially dependent on the subjectively selected input. Consequently, their analysis has no practical quantitative value.

Other sources of bias in their model include the failure to account for intentionally terminated pregnancies and the reduced fecundability of already pregnant women and nursing mothers. Despite this, it was described as “ *persuasive*” ^50^ and it has been claimed that “ *it is still difficult to better the original calculations of Roberts and Lowe (1975)*” ^27^. By contrast, others have noted that “ *their calculations can be criticized*” ^9^ and are “ *tenuous*” ^51^. Considering its quantitative limitations, it has been cited surprisingly often ^13, 28, 52, 53^.

### 2. Life tables of intrauterine mortality

**Figure 3.**
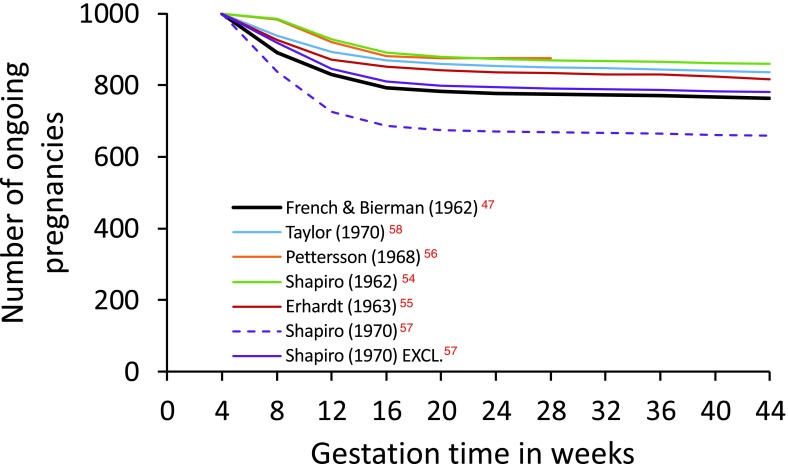
Graphical representation of the fate of 1,000 pregnancies in progress at 4 weeks’ gestation (2 weeks’ post-fertilisation). The figure is generated using values in Table 4.3 of Leridon (1977) ^26^ and are derived from six different studies (see text). The Kauai Pregnancy Study data published by French & Bierman (1962) ^47^ are shown in thick black. Data from Shapiro (1970) ^57^ were analysed either with all pregnancies included (ALL) or with those pregnancies excluded that aborted within one week of study entry (EXCL.). The greater loss observed with ALL may be due to a correlation between study entry and abortion risk. Based on these data, the risk of losing a pregnancy ongoing at 4 weeks’ gestation ranges from 12.5% to 23.7% (excluding Shapiro (1970) ALL). Values are in Dataset 2.

Constructing a life table of intrauterine mortality is challenging since embryonic death may occur even before the presence of an embryo is recognised. Nevertheless, in 1977, the distinguished demographer Henri Leridon published an impressive critique and analysis of pregnancy loss data, and a complete life table of intrauterine mortality ^26^. Leridon highlighted the consequences of inappropriate analysis and the quantitative biases produced by alternative numerical methods. Overall, he discussed sixteen studies, and provided detailed commentary on six published between 1962 and 1970 ^47, 54– 58^. These data are summarised in Figure 3 and suggest that 12–24% embryos alive at 4 weeks’ gestation (i.e., approx. 2 weeks’ post-fertilisation, see Figure 1) will perish before birth.

Figure 3 data See README.docx for a description of the file. Click here for additional data file. Copyright: © 2017 Jarvis GE 2017 Data associated with the article are available under the terms of the Creative Commons Zero "No rights reserved" data waiver (CC0 1.0 Public domain dedication).

Leridon described the Kauai Pregnancy Study published by French & Bierman (1962) ^47^ in particular detail. In this study, an attempt was made to identify every pregnancy on Kauai from 1953–56. Women were encouraged to enrol as soon as they missed a period. Pregnancy loss may therefore have been overestimated, since not all amenorrhoea is caused by conception, although other studies that relied upon medically-identified pregnancies probably underestimated pregnancy loss by not capturing all cases ^16^. Whatever the truth, it is clear that, among the studies reviewed by Leridon, the Kauai Pregnancy Study revealed the highest levels of pregnancy loss ( Figure 3).

All recorded pregnancies in the Kauai study were categorised by date of enrolment in four week intervals, beginning with 4–7 weeks’ gestation. This time-staggered approach enabled risk of miscarriage to be associated with stage of gestation. However, despite considerable efforts, only 19% of the 3,197 recorded Kauai pregnancies were enrolled between 4–7 weeks’ gestation, thereby reducing the precision of pregnancy loss estimates for this earliest of time intervals. Although pregnancies were grouped in four week periods, Leridon suggested that early mortality may change week by week, resulting in underestimation of pregnancy loss. He re-allocated the 592 study entries and 32 pregnancy losses for weeks 4–7 ( Table 2) generating an overall probability of pregnancy loss during this period of 15.0%, higher than 10.8% originally reported ^47^. Leridon’s own description of this interpolation as “ *risky*” can be illustrated by adjusting his re-allocation ^26^. Transferring just two of the pregnancy losses out of or into the first week results in estimates of the 4–7 week pregnancy loss of 10.9% and 19.1% respectively ( Table 2). The validity of adjusting Leridon’s re-allocation may be questioned. However, pregnancy loss in week 4–5 of the Kauai Study would manifest as a menstrual period delayed by up to one week. This is far from being a robust pregnancy diagnosis and in a different study ^57^, exclusion of pregnancy losses reported within one week of study entry resulted in substantially different loss probabilities ( Figure 3) suggesting a confounding correlation between entry and loss ^26^. Nevertheless, the re-allocation does reinforce a concern highlighted by Leridon, namely the uncertainty that affects the first probability. Clearly, these estimates of early loss should be treated with caution.

**Table 2.** A speculative numerical re-allocation of entries and pregnancy losses during weeks 4–7 in the Kauai Pregnancy Study (KPS) ^47^. Minor differences in the re-allocation of the earliest pregnancy losses have a substantial effect on the overall measure of pregnancy loss for that period. (Adapted from Table 4.2 in Leridon ^26^.)

Time period of gestation	New entries into study in each time period		Actual pregnancy losses in each time period		% pregnancy loss in each time period		Surviving pregnancies in each time period	
	Leridon’s re- allocation	KPS	Leridon’s re- allocation & [variants]	KPS	Leridon’s re- allocation & [variants]	KPS	Leridon’s re- allocation & [variants]	KPS
4–5	80	592	2 [0, 4]	32	5.0 [0.0, 10.0]	10.8	100 [100, 100]	100
5–6	120		6 [6, 6]		4.3 [4.3, 4.4]		95 [100, 90]	
6–7	180		10 [11, 9]		3.5 [3.9, 3.2]		91 [96, 86]	
7–8	212		14 [15, 13]		3.0 [3.2, 2.8]		88 [92, 83]	
4–8							85 [89, 81]	89.2
**% loss**							**15.0 [10.9, 19.1]**	**10.8**

A more fundamental problem is that these data offer no insight into the fate of embryos prior to the earliest possible point of clinical pregnancy detection. Leridon completed his life table with values from Hertig’s 1967 analysis ^5^. He concluded that among 100 ova exposed to the risk of fertilisation, 16 are not fertilised, 15 die in week one (between fertilisation and implantation), and 27 die in week two (between implantation and the time of the first missed period). After two weeks his life table follows the Kauai probabilities closely ending with 31 live births. Leridon’s table therefore indicates an embryo mortality of 50% (42/84) within the first two weeks after fertilisation and a total mortality of 63% (53/84) from fertilisation to birth.

Leridon’s account of intrauterine mortality has been widely cited. However, its accuracy depends entirely on the quality and interpretation of the data from Hertig ^5^ and French & Bierman ^47^. French & Bierman’s approach probably resulted in an overestimate of total pregnancy loss and is certainly imprecise in its estimate of embryo loss in the four weeks following the first missed menstrual period. The reliability of Hertig’s estimates of embryo loss in the two weeks following fertilisation is considered below.

### 3. Biochemical detection of pregnancy using hCG

Quantification of pregnancy loss requires pregnancy diagnosis. The earliest outward sign of pregnancy is a missed menstrual period, approximately 2 weeks after fertilisation, although amenorrhoea in women of reproductive age is not exclusively associated with fertilisation ^59, 60^. Several potentially diagnostic pregnancy-associated proteins have been identified ^61^ of which only one, Early Pregnancy Factor (EPF) ^62^, has been claimed to be produced by embryos within one day of fertilisation. However, there is doubt about the utility of EPF for diagnosing early pregnancy ^63^ and little has been published on it in the past five years.

Modern pregnancy tests detect human chorionic gonadotrophin (hCG), a highly glycosylated 37 kDa protein hormone produced by embryonic trophoblast cells ^64^. Elevation of hCG around 6–7 days after ovulation is associated with embryo implantation ^27, 28, 65^ ( Figure 1). Early assays for the detection of hCG were probably confounded by antibody cross-reactivity with luteinizing hormone ^66^ but modern tests are more specific and a positive result is a reliable indicator of early pregnancy. Highly sensitive assays have revealed low levels of hCG in non-pregnant women and healthy men ^67^; hence, quantitative criteria and appropriate design are required to distinguish between non-pregnant women and those harbouring early embryos ^65, 68, 69^.

**Figure 4.**
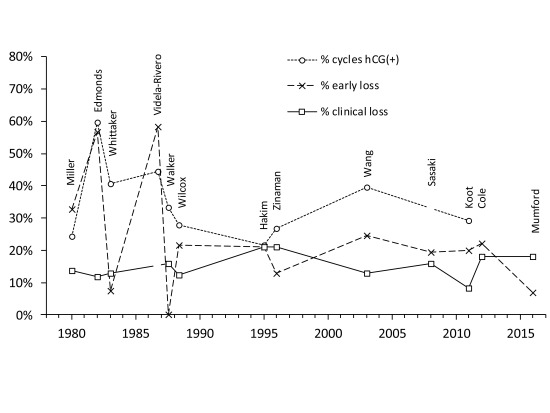
Summary of findings from thirteen studies that used hCG detection to diagnose early pregnancy. Data are arranged by publication date and the first author of the study is shown. Three datasets are shown: (i) the percentage of at risk menstrual cycles that were hCG positive; (ii) the percentage of hCG positive cycles that did not manifest as clinical pregnancies = early pregnancy loss; and (iii) the percentage of clinical pregnancies lost prior to 12 or 28 weeks or live birth (definitions vary between studies). A clinical pregnancy may be manifest by a missed period although criteria vary between studies. Videla-Rivero *et al.* ^73^, Sasaki *et al.* ^76^, Cole ^78^ and Mumford *et al.* ^79^ do not report sufficient data to calculate all three values. Values are in Dataset 3.

Figure 4 and Table 3 summarise findings from thirteen studies that used hCG to identify so-called early, occult or biochemical pregnancy loss, i.e., pregnancy loss between the initiation of implantation and clinical recognition ^46, 48, 49, 70– 79^. (Ellish *et al*. (1996) ^69^ is not included since the hCG assay was positive for only 72.5% of clinical pregnancies. By contrast, among the thirteen studies in Table 3 only one clinically-recognised pregnancy was reported undetected by hCG testing ^70^. Nevertheless, their estimates of early pregnancy loss (17.4%) and clinical loss (13.7%) are comparable to these other studies.) Each study measured urinary hCG levels except two, which measured hCG in serum ^72, 73^. Notwithstanding design and subject differences, estimates for clinical pregnancy loss, ranging from 8.3% – 21.2% ( Figure 4), are similar to previous estimates ( Figure 3). Estimates for early/occult pregnancy loss ranged from 0% to 58.3% in studies ^70– 74^ prior to Wilcox (1988) ^49^. This high variance was probably due to reduced specificity and sensitivity of the hCG assays and sub-optimal study design ^16, 61, 80– 83^. Studies from Wilcox (1988) ^49^ onwards have produced more consistent data indicating early/occult pregnancy loss of approximately 20% ( Figure 4). In the largest studies ^48, 79^ pregnancies were clinically recognised only if they lasted ≥6 weeks after the onset of the last menstrual period; hence, early pregnancy losses in these studies included those lost up to approximately two weeks after a missed menstrual period. Definition of clinical pregnancies can influence comparison of study results ^39, 82^. For example, Wilcox originally reported 43 (later 44 ^85^) pre-clinical and 19 clinical losses from 198 detected pregnancies ^49^, giving 21.7% early/occult and 12.3% clinical loss rates. In later reports, 4 cases were re-allocated resulting in 48 early and 15 clinical losses (i.e., 24.1% early/occult and 9.9% clinical loss rates) ^40, 86– 88^. Variable definitions can generate confusion, although in this case the overall picture is not greatly affected. Based on the eight most recent studies, beginning with Wilcox (1988), pregnancy loss from first detection of hCG through to live birth is approximately one third ( Table 3). This is consistent with another recent study which found that 98 out of 301 (32.6%) singleton pregnancies diagnosed by an early positive hCG test and followed-up to either birth or miscarriage were lost ^89^.

**Table 3.** Summary data from thirteen studies using hCG detection to diagnose pregnancy and identify early pregnancy loss. Raw *FEC _HCG_* is the ratio of hCG pregnancies detected and the number of cycles monitored in each study. Where available, mean (SD) ages of the participating women are taken directly from the published study. In some cases mean and SD (indicated by *) or SD (indicated by †) were estimated based on published demographic characteristics. ^§^These data relate to the whole study cohort (n=124) which included known sub-fertile women, and not just to the 74 apparently fertile women. ^‡^Mean value from Wilcox *et al*. (2001) ^84^. ^¶^Some studies only provide data up to late pregnancy (e.g., up to 28 weeks) rather than to term. ND = no data. ^¤^Wilcox subsequently reported an additional hCG pregnancy which had not been detected and reported in the 1988 paper, making a total of 199 hCG pregnancies and 44 pre-clinical losses in the study group ^85^. ^#^Mumford reported data from aspirin- and placebo-treated subjects who had at least one prior miscarriage. Summary data from both treatment groups are included as there was no effect of aspirin ^79^.

First author	Year	Number of women	Age mean (SD) [range]	Number of cycles	hCG pregnancies detected	Raw *FEC _HCG_*	Clinical pregnancies detected	% survival from hCG to clinical detection	% loss from hCG detection to live birth ^¶^
Miller	1980	197	27 (4)*	623	152	24.4%	102	67.1%	42.4%
Edmonds	1982	82	27 (4)*	198	118	59.6%	51	43.2%	61.9%
Whittaker	1983	91	30 (3.7) ^†^	226	92	40.7%	85	92.4%	19.6%
Videla- Rivero	1987	27	ND	27	12	44.4%	5	41.7%	ND
Walker	1988	38	27.4 [22–38]	75	25	33.3%	25	100%	16.0%
Wilcox	1988	221	30 ^‡^ (4)*	707	198 ^¤^	28.0%	155	78.3%	31.3%
Hakim	1995	74	31 (3)* ^§^	305	66	21.6%	52	78.8%	37.9%
Zinaman	1996	200	30.6 (3.3)	432	116	26.9%	101	87.1%	31.3%
Wang	2003	518	24.9 (1.7)	1,561	618	39.6%	466	75.4%	35.7%
Sasaki	2008	110	[21–36]	ND	62	ND	50	80.6%	32.3%
Koot	2011	46	28.7 (3.3)	103	30	29.1%	24	80.0%	26.7%
Cole	2012	168	28.8 (4.4)	ND	127	ND	99	78.0%	36.2%
Mumford	2016	1088 ^#^	28.7 (4.8)	ND	785	ND	730	93.0%	23.9%

Figure 4 data See README.docx for a description of the file. Click here for additional data file. Copyright: © 2017 Jarvis GE 2017 Data associated with the article are available under the terms of the Creative Commons Zero "No rights reserved" data waiver (CC0 1.0 Public domain dedication).

The much cited Wilcox (1988) study ^49^ is the earliest of several large well-designed studies that made use of a specific and sensitive hCG assay and led to numerous further publications ^40, 84– 87, 90– 92^. Two other studies (Zinaman (1996) ^46^ and Wang (2003) ^48^) were similar in purpose, design and execution. These studies provide some of the best available data to calculate pregnancy loss between implantation and birth ^39^. In each study, women intending to become pregnant and with no known fertility problems were recruited and hCG levels monitored cycle by cycle in daily urine samples until they became pregnant. Most women were followed through to late pregnancy or birth. Although these studies provide evidence regarding the outcome of both clinical and hCG pregnancies, determining the fate of embryos prior to implantation is more difficult. To relate the study results to pre-implantation embryo loss, it is necessary to determine fecundability. In each study *FEC _CLIN_* declined in successive cycles as the proportion of sub-fertile women increased. Hence, reported raw *FEC _HCG_* values of 30% ^46^ and 40% ^48^, and *FEC _CLIN_* values of 25% ^49^ and 30% ^48^ are biased underestimates of the fecundability of normal fertile women. A recent re-analysis of these data provides statistical evidence for discrete fertile and sub-fertile sub-cohorts within the study populations ^39^. The proportions of sub-fertile women (mean [95% CI]) were estimated as 28.1% [20.6, 36.9] (Wilcox); 22.8% [12.9, 37.2] (Zinaman); and 6.0% [2.8, 12.3] (Wang). For normally fertile women, *FEC _HCG_* was, respectively: 43.2% [35.6, 51.1]; 38.1% [32.7, 43.7]; and 46.2% [42.8, 49.6]. *FEC _CLIN_* was: 33.9% [29.4, 38.6]; 33.3% [27.6, 39.6]; and 34.9% [33.0, 36.8]. There was no apparent difference in *π _CLIN_* between fertile and sub-fertile sub-cohorts, which was estimated as: 78.3% [69.2, 85.3]; 87.5% [76.0, 93.9]; and 75.4% [71.5, 79.0] ^39^.

Why do a proportion of menstrual cycles in women attempting to conceive fail to show any increase in hCG? Since *FEC _HCG_* = *π _SOC_ × π _FERT_ × π _HCG_* , there can be various causes for this failure including mistimed coitus, anovulation, failure of fertilisation or pre-implantation embryo death. Although *FEC _HCG_* puts limits on the extent of pre-implantation embryo loss, uncertainty in the estimates of *π _SOC_, π _FERT_* and *π _HCG_* translates into uncertainty in estimates of pre-implantation embryo mortality. In the Wang study, for normally fertile women, *FEC _HCG_* = 46.2%; hence, the absolute maximum value for pre-implantation embryo loss must be 53.8%, although only if *π _SOC_* = *π _FERT_* = 1, conditions both extreme and unlikely ^39^. Studies of the relationship between coital frequency and conception indicate that fecundability is greater with daily compared to alternate day intercourse ^39, 93, 94^. Hence, when coital frequency is less than once per day a proportion of reproductive failure will be due to mistimed coitus, i.e., *π _SOC_* < 1. In the Wilcox study, coitus occurred on only 40% of the six pre-ovulatory days ^39, 40^, and in the Zinaman study participants were advised that alternate day intercourse was optimal ^46^. Based on the difference in fecundability between daily and alternate day intercourse as modelled by Schwartz ^94^, a value of *π _SOC_* = 0.80 was used to calculate pre-implantation embryo mortality ^39^. However, this is a speculative estimate, and in reality the value may be higher, or lower.

A further critical missing piece of the equation is knowledge of the efficiencies of fertilisation and implantation under normal, natural, propitious circumstances. Assuming that either of these processes may be up to 90% efficient, and based on data from the three hCG studies ^46, 48, 49^, a plausible range for pre-implantation embryo loss in normally fertile women is 10–40% and for loss from fertilisation to birth, 40–60% ^39^. Even with these wide ranges of mathematically possible outcomes, it is clear that estimates for total embryonic loss of 90% ^37^, 85% ^36^, 83% ^2^, 80–85% ^11, 35^, 78% ^34^, 76% ^10, 33^ and 70% ^27– 31^ are excessive.

In 1990, Charles Boklage concluded that “ *at least 73% of natural single conceptions have no real chance of surviving 6 weeks of gestation*” ^10, 95^. Live birth fecundability was estimated as “ *not over 15%*”, substantially lower than Leridon’s 31%. Despite this discrepancy, Boklage’s conclusions were derived from a review of data including several hCG studies ^49, 65, 70– 73^ and Leridon’s analysis ^26^. He derived a model describing the survival probability of human embryos comprising the sum of two exponential functions:

*P _t_*(pregnancy survival) = **0.73** *e* ^-0.155 *t*^ + 0.27 *e* ^-0.00042 *t*^

in which *t* is the time in days post-fertilization. This is the source of the 73% in the conclusion.

There are, however, serious problems with this analysis. Firstly, data presented as embryo survival probabilities at different times post-fertilization ^49, 65, 70, 71, 73^ are fecundabilities, i.e., successes per cycle, not per fertilised embryo. Secondly, for reasons that are unclear, data from Whittaker ^72^ and Leridon ^26^ were excluded from the modelling analysis and the data from an earlier Wilcox report ^65^ were included twice since this preliminary data had been incorporated into the later report ^49^. Thirdly, the modelled data were normalised to a survival probability of 0.287 at 21 days post-fertilization. This value was derived from data published by Barrett & Marshall (1969) on the relationship between coital frequency and conception ^93^. Barrett & Marshall had concluded that coitus during a single day alone, 2 days before ovulation, resulted in a conception probability of 0.30. Boklage’s value of 0.287 is his calculated equivalent. However, conception in this study was “ *identified by the absence of menstruation, after ovulation*” ^93^. Hence, 0.30 (and similarly, 0.287) is a clinical fecundability and not a measure of embryo survival. Furthermore, 0.30 is a non-maximal fecundability, since it was an estimate based on coitus on a single day (2 days before ovulation) within the cycle. Barrett & Marshall clearly report that as coital frequency increased so did the fecundability, up to a maximum of 0.68 associated with daily coitus ^93^.

Boklage’s analysis can only make biological sense if it is assumed that every cycle in the Barrett & Marshall study resulted in fertilisation. Under these circumstances, failure to detect conception in 71.3% (1 – 0.287) of cycles would be due entirely to embryo mortality. However, this is highly implausible and explicitly contradicted by the higher estimate of fecundability reported ^93^. Boklage’s implicit assumption also contradicts his further conclusion that “ *only 60–70% of all oocytes are successfully fertilized given optimum timing of natural insemination*” ^10^. The vertical normalisation of the hCG study data to a value of 0.287 at 21 days is the principal determinant of the parameters that define the two exponential model. Any change in this value would commensurately alter the balance between the two implied sub-populations of embryos. Since it is evident that the value of 0.287 is neither an embryo survival rate nor even a maximal fecundability, it follows that quantitative conclusions from this analysis in relation to the survival of naturally conceived human embryos are of doubtful validity.

However, Boklage was right about two things. Firstly, the difficulty of calculating pre-clinical losses: as he put it, “ *In the place of the necessary numbers for the first few weeks of pregnancy we find editorially acceptable estimates which, while perhaps not far wrong, are difficult to defend with any precision*”. Secondly, the source of some of the only directly relevant data (even though he excluded it from his modelling analysis), namely, “ *Hertig’s sample is, and will probably remain, unique*”.

### 4. The anatomical studies of Dr Arthur Hertig

At the start of the 1930s, no-one had ever seen a newly fertilised human embryo. It was barely 60 years since Oscar Hertwig had first observed fertilisation in sea urchins ^96^, and just 40 years before the birth in 1978 of Louise Brown, the first test tube baby ^97, 98^. In Boston, Dr Arthur Hertig and Dr John Rock’s search for early human embryos generated an irreplaceable collection, and set an influential benchmark for the scale of early human embryo mortality.

The so-called “Boston Egg Hunt” began in 1938 ^99^. Hertig and Rock recruited 210 married women of proven fertility who presented for gynaecological surgery ^44^. (In most of their publications, the number is given as 210 ^5, 100, 101^ although 211 subjects are mentioned elsewhere ^44^.) Of these, 107 were considered optimal for finding an embryo because they apparently: (i) demonstrated ovulation; (ii) had at least one recorded coital date within 24 hours before or after the estimated time of ovulation; (iii) lacked pathologic conditions that would interfere with conception. Hertig examined the excised uteri and fallopian tubes, and over fifteen years found 34 human embryos aged up to 17 days ^5, 44, 100– 107^. Of these, 24 were normal and 10 abnormal ^5, 100^. (There is some confusion over this: in three publications ^44, 101, 107^, 21 embryos are described as normal and 13 as abnormal. It appears that the three alternatively described embryos (C-8299; C-8000; C-8290) were originally defined as abnormal based on their position or depth of implantation ^44^.) Table 4 provides information about the 34 embryos found in these 107 women. Although the study was primarily intended to find and describe early human embryos, Hertig subsequently used the data to derive estimates of reproductive efficiency including early embryo wastage ^5, 100^.

**Table 4.** Summary of the characteristics of Hertig’s 34 embryos (values are taken from Figure 4 in Hertig *et al.* (1959)) ^100^. The embryos were collected from 107 out of 210 women. *In Hertig’s figure, day 28 of the ovulatory cycle is identified with day 1 of the next cycle and is the day of the presumed missed period in cases where pregnancy had commenced. The 36 cases that provide the evidential foundation for his numerical analysis are shown in bold.

Day of cycle	Biological description/ stage	Approx. age of embryos (days)	Number of cases	Embryos found	Normal embryos	Abnormal embryos	Detection rate (%)
14	Ovulation ± fertilisation	0	0	0	0	0	
16–17	Embryo suspended in fallopian tube	2–3	9	1	1	0	11.1%
18–19	Embryo suspended in uterus	4–5	15	7	3	4	46.7%
20–24	Implantation	6–10	47	5	5	0	10.6%
**25–3**	**First missed period on** day 28/1*	**11–16**	**36**	**21**	**15**	**6**	**58.3%**
Total			107	34	24	10	31.8%

Hertig’s analysis ^5, 100^ relies heavily on the 15 normal and 6 abnormal implanted embryos found in 36 women from cycle day 25 onwards. He assumed the 6 abnormal embryos would perish around the time of the first period concluding that fertility (% pregnant) at this stage = 42% (15/36). Of the 8 pre-implantation embryos identified (7 in the uterus and 1 in the fallopian tubes), 4 were abnormal. Hertig assumed the 4 normal embryos would implant successfully but that some of the abnormal ones would not, such that the proportion of normal embryos would increase from 50% (4/8) before implantation to 71% (15/21) after implantation as observed. Hence, among the 36 post-cycle day 25 cases, in addition to the 15 normal embryos, there must have been 15 abnormal pre-implantation embryos of which 60% (9/15) failed to implant and were not observed, and 40% (6/15) did implant and were observed, although these 6 would have perished shortly afterwards. This left 6/36 eggs that must have been unfertilised. The ratio of ‘unfertilised’: ‘fertilised abnormal’: ‘fertilised normal’ was therefore 6:15:15, matching the 16% infertility (no fertilisation), 42% sterility (post-fertilisation death) and 42% fertility (reproductive success) reported in Figure 9 of Hertig’s 1967 article, “ *The Overall Problem in Man*” ^5^. This is the source of Hertig’s 84% fertilisation rate and 50% embryo loss before and during implantation, and is reproduced in Leridon’s life table ^26^ as 84/100 eggs surviving at time zero (ovulation and fertilisation) and 42 surviving to 2 weeks (time of first missed period).

Hertig provides almost the entire body of evidence used to quantify natural human embryo loss in the first week post-fertilisation. Most claims regarding early human embryo mortality find their source here. Before considering how reliable the figures are, it is worth repeating Hertig’s own caveat, namely, the lack of data on the efficiency of natural fertilisation ^5^. All estimates of embryo mortality from fertilisation onwards are subject to commensurate inaccuracy in the absence of reliable fertilisation probabilities (i.e., *π _FERT_*), which are “ *surprisingly difficult to estimate*” ^21^.

There are several problems with Hertig’s analysis. As noted by others, the observations are cross-sectional, but the inferences are longitudinal ^108^. Hertig detected 21 embryos from 36 cases (58.3%) from cycle day 25 onwards. If this detection rate were representative, then on average, prior to day 25, the detection rate should either be the same or higher; however, they are all lower, and substantially so ( Table 4). Hertig suggested that this was due to the technical difficulty of finding newly fertilised embryos. However, the detection rate for cycle days 18–19 was good (46.7%) and embryos one or two days younger would not have been much smaller, at which stage the detection rate was poor (11.1%). An alternative explanation for this discrepancy might simply be random variation. Furthermore, from cycle day 25 onwards, embryos would probably have produced hCG and therefore *FEC _HCG_* would have been at least 58%. This is approximately double the equivalent values observed in more recent and robust hCG studies ( Table 3) further suggesting that this subset of the data is not representative.

Despite having proven fertility, these women presented for gynaecological surgery which, according to Hertig, was “medically essential” ^99^. This suggests that the women may have had sub-optimal reproductive function, although the effect of this on the quantitative outcome of the study is difficult to gauge. Furthermore, Hertig’s reproductively ‘optimal’ coital pattern does not include 2 days pre-ovulation and does include one day post-ovulation, conditions which are known not to maximise fertilisation ^39, 40, 93, 94, 109^. Hence, detection rates before cycle day 25 may be more representative than those after. Given the numerical discrepancies, they cannot both be.

Hertig does not provide error estimates with his conclusions. In order to estimate the precision of his derived proportions, a bootstrap analysis was performed as follows: Hertig’s 107 optimal cases were categorised according to stage of cycle (Category 1 = cycle days 16–19 (n=24); Category 2 = cycle days 20–24 (n=47); Category 3 = cycle days ≥25 (n=36)), and presence and type of embryos (Category 0 = no embryo (n=73); Category 1 = normal embryo (n=24); Category 3 = abnormal embryo (n=10)). Five hundred pseudo-datasets each containing 107 cases were generated using a balanced random re-sampling method using Microsoft Excel ^®^. The original and pseudo datasets are in Dataset 4.

Pseudo-datasets of Hertig’s study, obtained via a bootstrap procedure See README.docx for a description of the files. Click here for additional data file. Copyright: © 2017 Jarvis GE 2017 Data associated with the article are available under the terms of the Creative Commons Zero "No rights reserved" data waiver (CC0 1.0 Public domain dedication).

Hertig’s numerical calculations, as detailed above, were repeated for each pseudo-dataset thereby generating 500 estimates for each parameter, from which were derived median values and [95% CIs] using the percentile method 110: fertility = 42% [26%, 59%]; sterility = 42% [5%, 182%]; infertility = 16% [-127%, 61%]; pre-implantation embryo survival probability = 69% [27%, 128%]; post-implantation to week two survival probability = 71% [50%, 91%]; detection rate for cycle day 25 onwards = 58% [41%, 74%]. Median values matched estimates calculated from the original dataset. Bootstrap 95% CIs for the day 25 detection rate (58%) matched those calculated using the “exact” method of Clopper & Pearson ^111^, [41%, 74%], which are a little wider than those calculated using the “more exact” method of Agresti & Coull ^112^, [42%, 73%]. (These analyses were performed using an online GraphPad ^®^ calculator accessed on 18 ^th^ April 2017: http://www.graphpad.com/quickcalcs/ConfInterval1.cfm.) The congruence between these confidence intervals and the point estimates provides some reassurance that that the bootstrap procedure worked effectively. Estimates of parameters other than the day 25 detection rate (58%) are derived from more complex proportional relationships, and are therefore less precise. Table 5 reproduces a life table in the style of Leridon ^26^ and includes probabilities for each reproductive step with confidence intervals. These intervals (and some noted above) are impossibly wide highlighting further problems with Hertig’s analysis.

**Table 5.** Life Table of egg survival and probabilities during the first two weeks of development derived solely from Hertig’s data. The table is modelled on Leridon’s life table ^26^ and includes his values for survivors and data from Hertig ^5^. Probabilities are also shown for each stage of the early development process. Medians and 95% confidence intervals derived from a bootstrap analysis of Hertig’s data indicate the precision in the estimates for fertilisation and embryo loss in the first two weeks. *Although Leridon’s values are based on Hertig, they do not fully match. Leridon reports losses of 15 and 27 in the first and second weeks respectively. However, Hertig’s 60% loss of abnormal pre-implantation embryos implies 25 (0.6 × 42) losses in the first week leaving 58, and 16 (58 × (6/21)) losses in the second week, leaving 42. ^¥^A value of *π _SOC_* = 0.90 was used to avoid the calculation of probabilities greater than 1.

Week after Ovulation	Biological Description	Survivors (Leridon ^26^)	Survivors (Hertig ^5^)	Bootstrap Median [95% CIs]
	Number of Cycles	100	100	100 [100, 100]
0	Fertilised Eggs	84	83	84 [39, 227]
1	Implanted Embryos	69*	58	58 [41, 74]
2	Missed First Period	42	42	42 [26, 59]
**Probabilities**	**Biological Description**	**Probabilities**	**Probabilities**	**Bootstrap Median** **[95% CIs]**
*π _SOC_* × *π _FERT_*	Fertilisation per cycle	0.84	0.83	0.84 [0.39, 2.27]
*π _FERT_* (when *π _SOC_* = 0.90 ^¥^)	Fertilisation per ideal insemination	0.93	0.93	0.93 [0.43, 2.52]
*π _HCG_*	Fertilised egg implanting	0.82*	0.70	0.69 [0.27, 1.28]
*π _CLIN_*	Implanted egg to clinical recognition	0.61*	0.71	0.71 [0.50, 0.91]
*π _HCG_* × *π _CLIN_*	Fertilised egg to clinical recognition	0.50	0.50	0.50 [0.20, 0.88]

Hertig’s analysis omits 47 cases from cycle days 20–24, comprising 44% of his data. It is clear why he cannot use it, since all five embryos were normal and, given his mathematical and biological assumptions, five normal implanting embryos could not become 29% (6/21) abnormal post-implantation. Others have also noted that these “missing data are sufficient to engender an entirely different result” ^16^. Furthermore, the data that define the 50% proportion of abnormal pre-implantation embryos (i.e., 4/8) are so few that any numerical variation will make a substantial difference to derived proportions. If he had observed 3/8 abnormal embryos, his estimate of pre-implantation loss would have been 13% rather than 30%: for 5/8 it would have been 48%, with a fertilisation rate of 111%, which is clearly impossible. It seems therefore, that Hertig designed his analysis based on a post-hoc examination and selective use of the data. His own caveat about the lack of relevant and necessary data should be taken at least as seriously as his conclusions.

Hertig and Rock’s contribution to human embryology is undeniable and their quantitative conclusions have profoundly influenced our impression of the extent of early human embryo mortality. Regrettably, their estimates have a cripplingly low precision, which undermines their biological credibility or utility. In conclusion, Hertig’s data and flawed analysis cannot be regarded as a reliable quantitative foundation upon which to evaluate and understand natural human reproduction.

## Discussion

Answering the question “ *How many fertilised human embryos die before or during implantation under natural conditions?*” is difficult. Relevant, credible data are in short supply. Among regularly cited publications, the *Lancet* hypothesis ^34^ is entirely speculative and in the view of the current author should cease to be used as an authoritative source. Clinical pregnancy studies are only useful for quantifying clinical pregnancy loss and contribute nothing to estimates of embryo mortality in the first two weeks’ post-fertilisation. Even Hertig’s unique dataset is inadequate to draw quantitative conclusions and oft-repeated values should be treated with scepticism. The hCG studies from 1988 onwards provide the best data for estimating embryo mortality although a lack of information on fertilisation success rates ^5, 16, 21, 23, 113^ prevents satisfactory completion of the calculations. A recent re-analysis of these data proposed plausible limits for reproductively normal women indicating that approximately 10–40% of embryos perish before implantation and 40–60% do so between fertilisation and birth ^39^. However, these ranges are wide, particularly for pre-implantation mortality, reflecting the lack of appropriate data. Is there any possibility of narrowing down the numbers?

In the 1980s, two separate groups collected embryos from women following carefully timed artificial insemination as part of fertility treatment. Insemination around the time of ovulation in women of proven fertility was followed 5 days later by uterine lavage to recover ova ^114– 117^. These data appear to hold promise for determining fertilisation efficiency and some authors have made quantitative inferences about embryo mortality from them ^24, 27, 28^. However, such inferences are complicated by numerous confounding factors. For example, in one series ^116^, from 88 uterine lavages following artificial insemination by donor (AID), 4 unfertilised eggs, 6 fragmented eggs, and 27 embryos from 2 cell to blastocyst stage were retrieved. In the 51 cycles in which no egg or embryo was retrieved, there was one retained pregnancy suggesting that the lavage and ova retrieval efficiency was reasonably high, albeit not perfect. These data therefore suggest that *FEC _TOT_* was low (≈31/88 = 35%) although a proportion of fertilised eggs may have completely degenerated within the first 5 days. Assuming *π _SOC_* was high (given the targeted insemination), this suggests that *π _FERT_* ≈ 50%. In the context of the recent analysis ^39^, this implies that *π _HCG_* is high and that levels of embryo mortality are therefore towards the lower end of the 10–40% and 40–60% ranges. However, the clinical pregnancy rate following transfer of the embryos was only 40%. This is equivalent to *π _HCG_ × π _CLIN_*. If *π _CLIN_* ≈ 75%, as suggested by the hCG studies ( Table 3), this would mean that *π _HCG_* ≈ 50%. This would imply that *π _FERT_* is high, fertilised egg degeneration is high, occurs before day 5 and was therefore unobserved, and hence levels of embryo mortality tend towards the upper end of the 10–40% and 40–60% ranges.

It is possible that the lavage/transfer procedure reduced implantation and early developmental efficiency thereby reducing *π _HCG_ × π _CLIN_*. A comparison of AID pregnancy rates may provide some insight as suggested by the authors ^116^. The clinical pregnancy rate in their pharmacologically unstimulated cohort was 12.5% (11/88) which is lower than an equivalent 18.9% observed for fresh semen AID ^118^, and also the live birth rate (which also incorporates clinical pregnancy losses) of 14.7% reported by the HFEA for AID in 2012 in unstimulated women aged 18–34 ^119^. These different success rates suggest that the lavage/transfer procedure did adversely affect implantation and early gestation with clear implications for quantitative extrapolation. Furthermore, the women who were embryo recipients were receiving fertility treatment and their overall fertility may have been lower than expected in a normal healthy cohort. In summary, it seems that there are too many unresolved variables in these data to narrow down estimates of fertilization ( *π _FERT_*) or implantation ( *π _HCG_*) rates.

With high fecundability, the range of possible embryo mortality rates falls. Red deer hinds have pregnancy rates of >85% following natural mating ^120^: establishing numerical limits for embryo mortality under these efficient reproductive circumstances is more straightforward. By contrast, humans lack the instinct to mate predominantly during fertile periods thereby reducing observed reproductive efficiency substantially. In studies of early pregnancy loss, owing to sub-optimal coital frequency and cohorts including sub-fertile couples, natural fecundability was almost certainly not maximised ^39^. Combining data on coital frequency and hCG elevation may help to address this. In 1995, applying the Schwartz model ^94^ to his 1988 hCG data ^49^, Wilcox calculated a *FEC _HCG_* value of 36% for high coital frequencies (>4 days with intercourse in 6 pre-ovulatory days) ^40^. However, the Schwartz model assumed that cycle viability was evenly distributed among couples, a condition which the authors recognised was not true and is contradicted by a subsequent analysis which suggests that approximately a quarter of the Wilcox cohort was sub-fertile ^39^. If possible, focussing analytical attention on normally fertile women with the highest coital frequencies may help to narrow the range of plausible embryo mortality.

In this review of natural early embryo mortality no use has been made of data from *in vitro* fertilisation (IVF) and associated laboratory studies. Sub-optimal conditions for embryo culture mean that it was ^121, 122^ and probably still is ^123^ doubtful that reliable values can be extrapolated from laboratory *in vitro* to natural *in vivo* circumstances ^28^. Importantly, the reproductive stages are also altered. In IVF, *π _SOC_* = 1 and for transferred embryos *π _FERT_* = 1. Furthermore, transferred embryos are selected based on quality criteria, however inexact those may be ^123, 124^. IVF program manipulations may reduce *π _HCG_* compared to natural circumstances ^6^ and implantation failure remains a substantial issue for IVF ^125, 126^. Although for IVF cycles, the reported live birth rate per cycle has gone up (from 14% in 1991 to 25.4% in 2012 ^119^), comparison of IVF success rates and natural live birth fecundability values involves too many undefined variables to shed numerical light on early natural embryo development and mortality.

*In vitro* fertilisation *per se* may provide some insight into values of *π _FERT_*, since *π _SOC_* = 1, and successful fertilisation can be observed. In seven studies of natural cycle IVF, fertilisation was successful in 70.9% (443/625) of attempts ^127– 133^. If this represented natural, *in vivo* fertilisation, based on the recent analysis ^39^, it implies that *π _HCG_* ≈ 0.75, focusing estimates for pre-implantation embryo loss on 25%, and for total loss on 50%. However, high frequencies of chromosomal aberrations caused by the *in vitro* handling of human oocytes ^134^ can render any comparison of natural and assisted reproduction open to criticism ^9^.

In calculating summary values of embryo mortality, it is important to note that human fertility is as numerically heterogeneous as it could possibly be. Some couples are infertile and some are highly fertile. Excessive attention to averages and neglect of variances fosters a misleading appreciation of reality. The hCG studies clearly had both fertile and sub-fertile participants: use of overall values underestimated fecundability for the fertile majority ^39^. Furthermore, apparently ‘optimal’ conditions for conception may not maximise human biological fecundability. Other biological factors also contribute to reproductive heterogeneity in humans; however, even after controlling for age-related decline, fecundability remains highly variable ^119, 135^. For intercourse occurring 2 days prior to ovulation, average fecundabilities resembled those previously published ^88^, but for couples at the 5 ^th^ and 95 ^th^ percentiles, fecundabilities were 5% and 83%. 83% fecundability implies a very low embryo mortality rate. In conclusion, apparent low fecundability in humans need not necessarily be caused by embryo mortality, but also defects of ovulation, mistimed coitus, or fertilisation failure ^39^. Where fecundability is low, any or all of these factors may contribute.

Embryo mortality and pregnancy loss are not only a matter of academic scientific interest, and diverse quantitative estimates can also be found in popular media. For example, 70% loss in the first six days is claimed by Michael Mosley in “You made it through the first round” ( http://www.bbc.co.uk/timelines/z84tsg8; transcript at http://a.files.bbci.co.uk/bam/live/content/z3b87hv/transcript: accessed on 20 ^th^ April, 2017). By contrast a 25% pre-implantation loss is reported by the Science Museum’s online exhibit, “Who Am I?” ( http://www.sciencemuseum.org.uk/WhoAmI/FindOutMore/Yourbody/Wheredidyoucomefrom/Howdoyougrowinthewomb/Whathappensinweek1: accessed on 20 ^th^ April, 2017). News reports, often associated with ethical controversies, also feature estimates of embryo loss. On 1 ^st^ February 2016, James Gallagher reported that only 13/100 fertilised eggs develop beyond 3 months ( http://www.bbc.co.uk/news/health-35459054: accessed on 20 ^th^ April, 2017) and on 4 ^th^ May 2016, Sarah Knapton reported in the online *Daily Telegraph* that “two thirds of pregnancies fail because the embryo does not implant properly” ( http://www.telegraph.co.uk/science/2016/05/04/human-embryos-kept-alive-in-lab-for-unprecedented-13-days-so-sci/: accessed on 20 ^th^ April, 2017). In an ethical advocacy video, Bill Nye (“The Science Guy”) begins by claiming that “Many, many, many, many more hundreds of eggs are fertilized than become humans” ( https://www.youtube.com/watch?v=4IPrw0NYkMg: accessed on 20 ^th^ April, 2017). Additionally, academic philosophical articles ^1^ and legal judgements ^4^ have considered the significance of the scale of embryo loss. Given the breadth of societal interest in this facet of human reproductive biology, it is vital that scientists report plausible and defensible estimates for natural embryo mortality. Above all, it is obvious that women wishing to have children deserve reliable and unbiased estimates of reproductive success and pregnancy failure.

Pregnancy loss and embryo mortality under natural conditions are real and substantial. However, estimates of 90% ^37^, 85% ^36^, 80% ^11, 35^, 78% ^34^, 76% ^10, 33^ and 70% ^27– 31^ total loss are excessive and not supported by available data. Estimates for clinical pregnancy loss are approximately 10–20%. For women of reproductive age, losses between implantation and clinical recognition are approximately 10–25%. Loss from implantation to birth is approximately one third ^39, 46, 48, 49^.

Natural pre-implantation embryo loss remains quantitatively undefined. In the absence of knowledge of *π _SOC_* and *π _FERT_* it is almost impossible to estimate precisely. Hertig’s estimate is 30%; however, mathematically and biologically implausible confidence intervals [-28%, 73%] betray the quantitative weaknesses in his data and analysis. The best available data for quantifying early pregnancy loss are from studies monitoring daily hCG levels in women attempting to conceive. A recent re-analysis ^39^ of data from three studies ^46, 48, 49^ concluded that, in normal healthy women, 10–40% is a plausible range for pre-implantation embryo loss and overall pregnancy loss from fertilisation to birth is approximately 40–60%. This latter range is consistent with Kline's estimate of 50% ^16^, and similar to, although a little narrower than the 25–70% suggested by Professor Robert Edwards ^136^.

In the absence of suitable data to quantify pre-implantation loss, many published articles and reviews merely restate previously published values ^11, 28, 29^. It has been suggested that “ *claimed research findings may often be simply accurate measures of the prevailing bias*” ^137^. Widely held views on early embryo mortality may reflect an entrenched and biased view of the biology. For example, the Macklon “Black Box” review ^28^ has been cited 212 times (Web of Science ^TM^ citations on 12 ^th^ April 2017) with many articles explicitly referencing its 30% survival/70% failure values ^13, 29, 125, 138– 146^. Macklon’s quantitative summary in his “Pregnancy Loss Iceberg” (30% implantation failure; 30% early pregnancy loss; 10% clinical miscarriage; 30% live births) is a direct, unedited reproduction of estimates published over 10 years previously ^27^. 30% pre-implantation loss fairly represents Hertig’s conclusions although, as has been shown, this estimate is highly imprecise. However, Macklon misrepresents the best data which he reviews ^46, 49^. Wilcox reports early pregnancy loss (i.e., [1 - *π _CLIN_*]) of 21.7% whereas Macklon’s iceberg implies that 43% (30/70) of implanting embryos fail before clinical recognition. The iceberg’s clinical loss rate of 25% (10/40) is also higher than relevant data indicate ( Figure 3 & Figure 4). Total loss of implanting (hCG+) embryos (i.e., [1 - ( *π _CLIN_ × π _LB_*]) is 57% (40/70) according to the iceberg. By contrast, Wilcox ^49^ and Zinaman ^46^, both included in Macklon’s review, report that only 31% of hCG positive pregnancies fail.

Early pregnancy loss of 10–25% is not trivial. Despite difficulties associated with extrapolating *in vitro* observations to *in vivo* circumstances, implantation is clearly a crucial biological milestone in embryonic development and pregnancy. Recent studies suggest that biological mediators from embryos may regulate endometrial receptivity resulting in selective implantation of fitter embryos ^18, 147, 148^. Interestingly, supernatant from developmentally impaired IVF embryos deemed unsuitable for transfer provoked a different response in endometrial cells compared to supernatant from developmentally competent embryos that produced an ongoing pregnancy after transfer ^18^. However, it would be of interest to compare these responses to that provoked by supernatant from developmentally competent embryos that did not successfully implant and produce an ongoing pregnancy. Arrested or developmentally impaired embryos may lose cellular integrity and release mediators that disrupt endometrial receptivity; however, the intrinsic developmental competence of such embryos must also determine the success of implantation. Implantation failure is a substantial issue for IVF ^125^, and distinguishing between obviously impaired and competent embryos is not the principal challenge, but rather distinguishing from among apparently competent embryos those that will successfully implant and those that will not. Striking evidence suggests that only 9% of IVF embryos have a normal karyotype in all their cells ^145, 149^ raising the possibility that the rate of aneuploidy in IVF embryos is artefactually high, or that a degree of mosaic aneuploidy in human embryos is not necessarily developmentally deleterious, or indeed both. Implantation failure of a normal embryo may well be an uncommon event ^51^, but defining what is normal is a considerable scientific and conceptual challenge.

If Macklon’s ^28^ (and Chard’s ^27^) 70% estimate for embryo loss is excessive, as the data suggest, this casts doubt on claims ^125, 143^ that the frequency of embryonic abnormalities observed *in vitro* is representative of the natural *in vivo* situation. In turn, this implies that many of the chromosomal abnormalities observed in *in vitro* human embryos may be, to a greater extent than currently recognised ^125^, an artefact of the clinical and experimental interventions of assisted reproductive technologies.

This is not the first time that attention has been drawn to unsatisfactory estimates of early embryo loss. Faced with some of the same data, others have noted that “ *a claim of ‘no significant difference’ might easily be sustained against any interpretation proffered*” ^16^ and that estimates are “ *difficult to defend with any precision*” ^10^. Conclusions have been based on “ *poor estimates of fertilization failure rate and the mortality at 2 weeks after fertilisation*” ^23^ and drawn “ *from unusual or biased samples*” ^150^. Nevertheless, although precision may be elusive, exaggeration can be avoided. It is hoped that this critical re-evaluation of the data describing early human embryo mortality will serve as a robust foundation upon which to make informed biological, ethical, legal and personal judgements.

## Data availability

The data referenced by this article are under copyright with the following copyright statement: Copyright: © 2017 Jarvis GE

Data associated with the article are available under the terms of the Creative Commons Zero "No rights reserved" data waiver (CC0 1.0 Public domain dedication). 

*F1000Research*: Dataset 1. Figure 2 data, 10.5256/f1000research.8937.d140569 ^151^

*F1000Research*: Dataset 2. Figure 3 data, 10.5256/f1000research.8937.d140570 ^152^

*F1000Research*: Dataset 3. Figure 4 data, 10.5256/f1000research.8937.d140571 ^153^

*F1000Research*: Dataset 4. Pseudo-datasets of Hertig’s study, obtained via a bootstrap procedure, 10.5256/f1000research.8937.d140572 ^154^
