# Supplementary material for: Early embryo mortality in natural human reproduction: What the data say
Source: F1000Res. 2017 Jun 7;5:2765. Originally published 2016 Nov 25. [Version 2] doi: 10.12688/f1000research.8937.2 (PMC5443340; doi:10.12688/f1000research.8937.2)
Supplement: Figure 2 data — See README.docx for a description of the file. [file f1000research-5-12388-s0000.tgz › b40bc287-7c9e-49b4-820b-9f0bb2249e4c_Fig1_data.zip!/README.docx]

**Key for Dataset_1.csv**

\# is the index number for a simulated data record (total number = 100,000)

Original values from Roberts & Lowe, 1976 (RL)

- PCOIT is the RL estimate for the number of coital acts per week
- PUNPR is the RL estimate for the proportion of coital acts that are unprotected
- PFPER is the RL estimate for the number of days in a cycle that are fertile
- PCYCL is the RL estimate for the mean cycle length in days
- PFERT is the RL estimate for the fertilisation rate

Alternative randomly selected estimates

- ICOIT is an estimate for the number of coital acts per week, randomly sampled from *N*(μ = PCOIT, σ = 0.2 × PCOIT)
- IUNPR is an estimate for the proportion of coital acts that are unprotected, randomly sampled from *N*(μ = PUNPR, σ = 0.2 × PUNPR)
- IFPER is an estimate for the number of days in a cycle that are fertile, randomly sampled from *N*(μ = PFPER, σ = 0.2 × PFPER)
- ICYCL is an estimate for the mean cycle length in days, randomly sampled from *N*(μ = PCYCL, σ = 0.2 × PCYCL)
- IFERT is an estimate for the fertilisation rate, randomly sampled from *N*(μ = PFERT, σ = 0.2 × PFERT)

Alternative calculated values

- COIT# is the calculated number of coital acts per year for simulated data entry # (= 2,437,000 × 52 × ICOIT)
- UNPR# is the calculated number of unprotected coital acts per year for simulated data entry # (= COIT# × IUNPR)
- FPER# is the calculated proportion of each cycle that is potentially fertile for simulated data entry # (= IFPER ÷ ICYCL)
- COIF# is the calculated number of unprotected coital acts within a fertile period per year for simulated data entry # (= UNPR# × FPER#)
- FERT# = the calculated number of fertilisations per year for simulated data entry # (= COIF# × IFERT)
- LOSS# = the calculated proportion of embryos that are lost from fertilisation to live birth for simulated data entry # (= 1 – (505,000 ÷ FERT#)

```
; Code for NONMEM simulation (Figure 1)
; Each simulation generates 10,000 values
; The simulation was repeated 10 times with different random seed values
; See below for seed details

$PROBLEM Roberts & Lowe Embryo Mortality simulation
$DATA ..\Rob_Low_SIM1.CSV      IGNORE=C
$INPUT
  ID               ; record ID (1-10,000)
  DV               ; %loss     (all set to 0 for simulation)
$PRED
  pCOIT = THETA(1)       ; population acts of coitus per week
  pUNPR = THETA(2)       ; population P of unprotected coitus
  pFPER = THETA(3)       ; population # of fertile days per cycle
  pCYCL = THETA(4)       ; population # of days per cycle
  pFERT = THETA(5)       ; population P of fertilisation after coitus in fertile period
  iCOIT = pCOIT + ETA(1) ; individual acts of coitus per week
  iUNPR = pUNPR + ETA(2) ; individual % of unprotected coitus
  iFPER = pFPER + ETA(3) ; individual # of fertile days per cycle
  iCYCL = pCYCL + ETA(4) ; individual # of days per cycle
  iFERT = pFERT + ETA(5) ; individual P of fertilisation after coitus in fertile period
  MW    = 2437000        ; Number of Married women aged 20-29 in 1971
  AB    =  505000        ; Actual births to MW in 1971
  COIT  = MW * iCOIT * 52; Total Annual Acts of Coitus
  UNPR  = COIT * iUNPR   ; Total Annual Acts of Unprotected Coitus
  FPER  = iFPER / iCYCL  ; Proportion of time fertile
  COIF  = UNPR * FPER    ; Total Annual Acts of Unprotected Coitus in fertile period
  FERT  = COIF * iFERT   ; Total Annual number of successful fertilisations
  LOSS  = (FERT-AB)/FERT ; Proportion of conceptuses lost
$THETA  2    0.25   2    28    0.5  ; R&L estimates
$OMEGA  0.16 0.0025 0.16 31.36 0.01 ; R&L variances
$SIGMA  0 FIX
$SIM    (889215690) ONLYSIM PREDICTION ; Seed number changed for each simulation
$TABLE  ID DV
        pCOIT pUNPR pFPER pCYCL pFERT
        iCOIT iUNPR iFPER iCYCL iFERT
        COIT  UNPR  FPER  COIF  FERT  LOSS
        ONEHEADER NOPRINT FORMAT=s1PE17.9
        FILE=Rob_Low_SIM1.fit

; SIM seeds
; SIM1  889215690
; SIM2  889215691
; SIM3  889215692
; SIM4  889215693
; SIM5  889215694
; SIM6  889215695
; SIM7  889215696
; SIM8  889215697
; SIM9  889215698
; SIM10 889215699
```
