# Supplementary material for: Early embryo mortality in natural human reproduction: What the data say
Source: F1000Res. 2017 Jun 7;5:2765. Originally published 2016 Nov 25. [Version 2] doi: 10.12688/f1000research.8937.2 (PMC5443340; doi:10.12688/f1000research.8937.2)
Supplement: Figure 3 data — See README.docx for a description of the file. [file f1000research-5-12388-s0001.tgz › c74e5f66-b185-4b19-90a3-5f56de41d412_Fig2_data.zip!/README.docx]

**Key to Dataset_2.csv which contains the values for Figure 2.**

Values in Data Columns 2 – 8 represent the number of pregnancies ongoing at various stages of pregnancy, starting with 1,000 at week 4 of gestation.

The values are derived from: Leridon, H. *Human Fertility: The Basic Components*, Chapter 4, Intrauterine Mortality, Table 4.3 (p. 58-59). University of Chicago Press, Chicago (1977).

| **Data column** | **Column header** | **Description** |
| --- | --- | --- |
| 1 | Week | Gestational week. Time since last menstrual period. |
| 2 | French & Bierman (1962) | Reference 43 |
| 3 | Taylor (1970) | Reference 48 |
| 4 | Pettersson (1968) | Reference 46 |
| 5 | Shapiro (1962) | Reference 44 |
| 6 | Erhardt (1963) | Reference 45 |
| 7 | Shapiro (1970) ALL | Reference 47. Include ALL values |
| 8 | Shapiro (1970) EXCL. | Reference 47. Values exclude pregnancies ending in abortion the same week that the pregnancy entered the study |
