# Supplementary material for: Early embryo mortality in natural human reproduction: What the data say
Source: F1000Res. 2017 Jun 7;5:2765. Originally published 2016 Nov 25. [Version 2] doi: 10.12688/f1000research.8937.2 (PMC5443340; doi:10.12688/f1000research.8937.2)
Supplement: Figure 4 data — See README.docx for a description of the file. [file f1000research-5-12388-s0002.tgz › 7c8dc5e0-3437-4dc4-8c0c-f0f1a0b3a920_Fig3_data.zip!/README.docx]

**Key to Dataset3.csv which contains the values for Figure 3.**

Blank cells indicate no reported value

| **Data column** | **Column header** | **Description** | **Derivation** |
| --- | --- | --- | --- |
| 1 | No | Study number | Defined |
| 2 | Author | First Author of published study | Published |
| 3 | PMID | PubMed ID of published study | PubMed |
| 4 | Year | Year of publication | Published |
| 5 | x axis | Value of x-axis of Figure | Year of publication, modified to facilitate presentation of studies with the same year |
| 6 | % cycles hCG(+) | Values plotted in Figure | "hCG(+) cycles" / "ovulatory cycles" |
| 7 | % early loss | Values plotted in Figure | "EPL" / "hCG(+) cycles" |
| 8 | % clinical loss | Values plotted in Figure | "clin loss" / "clin preg" |
| 9 | ovulatory cycles | Number of ovulatory cycles reported by study | Published |
| 10 | hCG(+) cycles | Number of hCG(+) cycles reported by study | Published |
| 11 | EPL | Early pregnancy loss. The number of hCG(+) cycles that did not progress to a clinical pregnancy | Published |
| 12 | clin loss | Total clinical losses in study | "spont abort" + "ect/molar " + "still birth" |
| 13 | clin preg | Total clinical pregnancies in study | "hCG(+) cycles" - "EPL" - "TOP" - "ongoing" - "LFU" |
| 14 | spont abort | Number of spontaneous abortions and miscarriages of clinical pregnancies | Published |
| 15 | ect/molar | Number of ectopic or molar pregnancies among clinical pregnancies | Published |
| 16 | still birth | Number of still born infants | Published |
| 17 | TOP | Number of termination of pregnancies / therapeutic abortions | Published |
| 18 | ongoing | Number of ongoing clinical pregnancies | Published |
| 19 | LFU | Number of clinical pregnancies Lost to follow-up | Published |
