# Supplementary material for: Early embryo mortality in natural human reproduction: What the data say
Source: F1000Res. 2017 Jun 7;5:2765. Originally published 2016 Nov 25. [Version 2] doi: 10.12688/f1000research.8937.2 (PMC5443340; doi:10.12688/f1000research.8937.2)
Supplement: Pseudo-datasets of Hertig’s study, obtained via a bootstrap procedure — See README.docx for a description of the files. [file f1000research-5-12388-s0003.tgz › 263e7816-d3e9-4aff-97f8-32772f3b526d_Dataset_4.zip!/README.docx]

**Dataset 4 information**

Dataset 4 contains the pseudo-datasets of Hertig’s study, obtained via a bootstrap procedure. The data file contains the following.

**Column A**: Set 0: Index numbers for the original 107 optimal cases from Hertig

**Column B**: Cycle: Indicates at what stage of the reproductive cycle each case was from: 1 = days 16-19 (n=24); 2 = days 20-24 (n=47); 3 = cycle days ≥25 (n=36).

**Column C**: Embryo: Indicates what category of embryo was found in each case: 0 = no embryo (n=73); 1 = normal embryo (n=24); 2 = abnormal embryo (n=10).

**Columns D – SI**: Set0 - Set500. 500 pseudo-datasets listing the index numbers of the cases (n=107) randomly sampled from the original data set.
