# Supplementary material for: Early embryo mortality in natural human reproduction: What the data say
Source: F1000Res. 2017 Jun 7;5:2765. Originally published 2016 Nov 25. [Version 2] doi: 10.12688/f1000research.8937.2 (PMC5443340; doi:10.12688/f1000research.8937.2)
Supplement: Peer review history

# Peer review: Referee response for version 1

**DOI:** 10.5256/f1000research.9616.r22945

**Published:** 2017-05-23

**Authors:** James E. Zuckerman, Steven H. Orzack

**Affiliations:** 1 Brigham and Women's Hospital, Harvard Medical School , Boston, MA, USA; 2 Fresh Pond Research Institute, Cambridge, MA, USA

Dr. Jarvis assesses the empirical support for the belief that there is a “great deal” of fetal wastage in humans. His conclusion is that there is less wastage than is often believed and that the percent loss between conception and birth is 40-60%. Resolution of this issue is important, as it has substantial implications for our understanding of early human development.

 Dr. Jarvis describes present understanding as (p. 2):

*Among reputable scientific publications, including medical and reproductive biology text books, scientific reviews and primary research articles, reported mortality estimates include: 30–70% before and during implantation; >50%, 73% and 80% before the 6th week; 75% before the 8th week; 70% in the first trimester; 40– 50% in the first 20 weeks; and 49%, >50%, 53%, 54%, 60%, >60%, 63%, 70%, 50–75%, 76%, 78%, 80–85%, >85%, and 90% total loss from fertilisation to term.*

 He states (p. 2) that four types of evidence underlie these claims:

1. A speculative hypothesis published in The Lancet.
2. Life tables of intra-uterine mortality.
3. Studies of early pregnancy by biochemical detection of hCG.
4. Anatomical studies of Dr Arthur Hertig and Dr John Rock.

On the basis of his review of this evidence, Dr. Jarvis concludes (p. 12) that “….10-40% is a plausible range for pre-implantation embryo loss and overall pregnancy loss from fertilization to birth is approximately 40-60%.” 

 This means that the best estimate of pre-birth mortality according to Dr. Jarvis is consistent with many previous estimates. In order to understand this consistency, it is useful to examine these types of evidence and what Dr. Jarvis makes of each. I discuss them in turn.

**1. **The Lancet article is Roberts & Lowe (1975). These authors concluded (p. 498) from their “speculative” analysis of the number of married women age 20-29 in England and Wales and of the number of live and dead births that 78% of conceptions are lost. In order to generate this estimate, the authors estimated the number of conceptions in any given year (based on the number of sexual encounters, probability of fertilization, etc.). Dr. Jarvis assesses the influence of changing the number of conceptions on the estimate of fetal wastage and shows (p. 3) that a low estimate of the number of conceptions results in an estimate of 22% conceptions lost and that a high estimate of the number of conceptions results in an estimate of 92% loss. He also generates a 95% confidence interval for the loss percentage of 37% - 90% by doing a simulation in which each value contributing to the number of conceptions is normally-distributed with a mean identical to Roberts and Lowe’s value and a coefficient of variation of 20%. On this basis, he concludes about Roberts and Lowe’s analysis that (p. 1) it “….has no quantitative value.” and that (p. 4) it “….has no practical quantitative value”.

 Dr. Jarvis provides a useful sensitivity analysis of Roberts and Lowe’s estimate, which should be taken seriously by those who may believe that their analysis is definitive (their paper has been cited more than 300 times, with many citations that point to the 78% estimate). That said, Dr. Jarvis’ conclusion that Roberts and Lowe’s analysis is quantitatively useless is itself incoherent. A number is a number and as a starting point, their estimate is useful although limited. If their analysis lacks “practical quantitative value” so too does the analysis of Dr. Jarvis. After all, there is no empirical basis for his assumptions about the statistical independence of the components contributing to his estimate of percentage or that these components are normally-distributed or that they have a coefficient of variation of 20%. It is not as though simply making arbitrary assumptions about the variability of parameters somehow means that an analysis is more quantitatively useful than one without such assumptions. The point is that both analyses have value. It is telling in this regard that their estimate is “close” to Dr. Jarvis’ estimate. In fact, one could readily claim that Dr. Jarvis’s analysis validates Roberts and Lowe’s estimate in as much as their estimate is within the 95% confidence interval he generates.

 By way of understanding Robert and Lowe’s self-described “speculative” work, it is important to note it belongs to the voluminous “gray” literature relating to human pregnancy. This is the literature that is published without much review (if any) and without much requirement for rigor and data. To see this, one need go farther than this passage (p. 498):

*Animal studies, which allow a more systematic investigation of [pregnancy loss], have shown detectable prenatal losses ranging from 15 to 60% in domestic cattle, sheep, and pigs and in wild forms such as stoats, rats, squirrels, and rabbits.*

 They cite Austin (1972) for this claim. He merely states (p. 134): 

*The data show that prenatal losses ranging between 15 and 60 per cent occur in cattle, sheep and pigs, as well as in wild forms such as stoats, rats, squirrels and rabbits.*

 No data are cited! In fact, Austin’s gloss on the loss percentage for domesticated species is reasonably accurate (Casida, 1953; First & Eyestone, 1988; Lasley, 1957) although there are less data than one might imagine. It is of note that these species have been selected for offspring production and so how relevant these data are is not completely resolved. Perhaps fetal wastage in their wild relatives would be greater. My guess is that the data alluded to as being from “wild forms” are in papers such as those by Brambell (1942, 1948). That said, to my knowledge, it is not clear that such studies reliably account for early gestational losses. More generally, there are few “wild forms” for which there are estimates.

 The overall point is that Robert and Lowe’s paper contains a disconnection between data and conclusions that would be sustained even if one read the cited source. Their paper is best viewed as a heuristic exercise. This is not a criticism. It is meant to underscore that Dr. Jarvis’ conclusion that their paper is “useless” treats it as something that it isn’t. We are ignorant of the training of Drs. Robert and Lowe but like many authors of the gray literature concerning pregnancy, they may have lacked rigorous training in research practice and data analysis. This is not inherently bad, as long as the nature of such publications is properly understood. As a community of scientists, we can make use of their insight into human pregnancy as long as its potential limitations are understood. We need all the help we can get!

**2. **The “life tables of intra-uterine mortality” are French & Bierman (1962) and Léridon (1977).The former study is an analysis of pregnancies in Kauai, Hawaii; the authors’ conclusion was that approximately 24% of the pregnancies registered with an estimated gestational age of greater than four weeks would die. Léridon married this result with the data of Hertig, Rock, Adams, & Menkin (1959), which provide an estimate of wastage prior to four weeks, to infer that 63% of conceptions die before birth (Table 4.20, p. 81). Dr. Jarvis’ cautions about the assumptions that underlie this estimate are reasonable. That said, it is important to note that the Léridon’s chapter (“Intrauterine Mortality”, pp. 48-81) is no casual exercise. It is the longest chapter in the book and an open-minded reader can see that Table 4.20 is based upon reasonable assumptions that Léridon clearly states do not have as much of a solid empirical basis as would be desired. Unfortunately, Dr. Jarvis’ sole mentions of Léridon’s caveats are a statement (p. 5) in which

 Léridon describes (p. 56) an interpolation he makes (in his analysis of French and Bierman’s data) as “risky” and another in which his (Dr. Jarvis) reanalyses of the French and Bierman data (p. 5) “reinforce a concern highlighted by Léridon”. To this extent, a reader of Dr. Jarvis’ paper could easily come away with the mistaken belief that Léridon’s analysis is superficial at best. As in the case of Roberts and Lowe’s estimate, it is important to note that Léridon’s estimate of conceptions lost of 63% is close to Dr. Jarvis’ estimate of 40-60%.

**3. **“Studies of early pregnancy by biochemical detection of hCG.” The modern pregnancy test is based upon an assay of human chorionic gonadotrophin (hCG), an oligosaccharide glycoprotein hormone produced by embryonic cells. An elevated level of hCG is detectable six to fourteen days post-conception (Nepomnaschy, Weinberg, Wilcox, & Baird, 2008; Wilcox, Baird, & Weinberg,1999). By this time, most embryos capable of implantation will have done so. Unfortunately, earlier pre-implantation detection of pregnancy based upon assay of the “Early Pregnancy Factor”, a heat-shock protein expressed within 48 hours of conception, is not in widespread use (Clarke, 1997; Fan & Zheng, 1997; Morton, Rolfe, & Cavanagh, 1992; Rolfe, 1982; Shahani, Moniz, Chitlange, & Meherji, 1991; Shahani, Moniz, Gokral, & Meherji, 1995; Smart, Fraser, Roberts, Clancy, & Cripps, 1982). Dr. Jarvis correctly describes the pioneering hCG results of Wilcox *et al.* (1988) and others (as summarized in Table 3), which indicate that the percentage loss of conceptions after hCG detection is between approximately 20 and 60%, with many estimates between 30 and 40%; Dr. Jarvis concludes (p. 6) that this percentage loss is approximately 33%.

 Dr. Jarvis goes on to estimate that the “…loss from fertilization to birth [is] 40- 60%”; this is based on the combination of three estimates based on hCG assay of percentage loss from conception to birth (35.7%: Wang *et al.*, 2003; 31.3%: 31.3%: Wilcox *et al.*, 1988; 31.3% Zinaman, Clegg, Brown, O’Connor, & Selevan, 1996) and his estimate (pp. 7-8) that the efficiency of implantation of embryos “…may be up to 90% efficient….” in order. He concludes that higher estimates of loss from fertilization to birth from the literature are “excessive”.

 Dr. Jarvis’ estimate is likely an underestimate. There is strong circumstantial evidence that many more than 10% of embryos do not successfully implant, as discussed below. The implication of this is that Dr. Jarvis’ estimate and the previous estimates are consistent. It is also worth noting that Dr. Jarvis uses an arbitrary estimate for implantation rate, even though he judges other analyses to be useless because they contain an arbitrary parameter estimate. 

 Dr. Jarvis goes on to criticize Boklage (1990) who estimated the percentage of unsuccessful conceptions based on an analysis of hCG data (see his Figure 2, p. 84). Dr. Jarvis is right to raise concerns (p. 8) that Boklage’s analysis is less definitive than desired. In particular, he states (p. 8) that Boklage’s assumption that the 21-day survival rate of conceptions is 28.7% is based upon a misinterpretation of a previous study. That said, Dr. Jarvis makes an unsubstantiated conclusion (p. 8) that “…quantitative conclusions from [Boklage’s] analysis in relation to the survival of naturally conceived human embryos are of doubtful validity”. This may be true, but this remains to be seen given the lack of any demonstration of the sensitivity of Boklage’s quantitative conclusions to changes in the underlying assumptions. Boklage’s analysis needs more careful scrutiny than given by Dr. Jarvis. For example, Boklage presents a formula for the percentage loss of conceptions as a function of time (p. 84). Are the coefficients estimated via a standard statistical approach such as maximum likelihood estimation and chosen via a likelihood ratio test or via comparison of AIC values associated with competing models? This is not clear. As such, it is unclear as to what to make of the predictions even putting aside Dr. Jarvis’ concerns about the biological validity of some of the underlying data. The equation appears to be based upon the assumption that a cohort of embryos is an admixture of those that are likely to die before six weeks and those that will survive longer. The basis for this assumption is unclear. The lack of transparency of Boklage’s equation is underscored by the fact that Dr. Jarvis does not mention that it predicts 75.8 percent fetal wastage between conception and full-term birth (270 days). As above, this estimate is rightly or wrongly consistent with most

 previous estimates.

**4. **The “anatomical studies of Dr Arthur Hertig and Dr John Rock” are investigations of conceptions recovered from uteri obtained via gynecologic surgery. Their results are summarized in Hertig *et al. *(1959); Hertig & Rock, (1973); Hertig, (1967). As described by Dr. Jarvis (p. 9), Hertig *et al.*’s conclusion is that 50% of embryos will die within two weeks after conception. 

 Dr. Jarvis’ is correct to point out concerns about their conclusion, although we believe that it has been well recognized that it is “impressionistic” as opposed to something that has a solid quantitative underpinning. Of course, as noted by Dr. Jarvis, their work remains important. 

 Dr. Jarvis makes some assertions about Hertig *et al.*’s work that seem mainly intended to accentuate doubts about it as opposed to placing it in proper context. He notes correctly (p. 9) that the sample is cross-sectional and not longitudinal. Given the nature of this study, this was unavoidable. Dr. Jarvis notes there are some unresolved discrepancies among age-specific detection rates for embryos and also between the estimated implantation rate and the rate inferred from other studies. These are worth mentioning but the implications of these discrepancies remain ambiguous in the absence of a quantitative analysis that accounts for sampling variation.

 Similarly un-useful is Dr. Jarvis’ statement (p. 9) that “Despite having proven fertility, these women presented with gynaecological problems, suggesting suboptimal reproductive function.” There is a wide range of “gynaecological problems” and an unanchored assertion that such a broad category might result in “sub-optimal reproductive function” means nothing in the absence of evidence that whatever problems were present had some influence on embryonic viability. In an effort to “estimate the precision” of the various proportions presented by Hertig *et al.* (e.g., the survival rate to implantation), Dr. Jarvis generated 500 so called “bootstrap” samples from the original data consisting of 107 cases. These samples arise from sampling with replacement of the original data (e.g., see Efron & Tibshirani, 1986; Efron, 1987). Such an investigation is worthwhile, although a bootstrap analysis is not a “cure” for small sample size. In any case, Dr. Jarvis’ analyses of the bootstrap results are incorrect. He describes (p. 10) “95% CIs” for various proportions that are outside of the range of 0-100%. For example, the confidence interval (p. 10) he provides for pre-implantation embryo survival probability is 27-128%. Such an interval cannot be generated by a correct bootstrap analysis. There are various ways to calculate a bootstrap confidence interval (Efron & Tibshirani, 1986). The simplest, known as the “percentile method”, generates a 95% bootstrap confidence interval for a proportion directly from the range of proportions associated with the central 95% of the bootstrap estimates. Accordingly, the confidence interval must be between 0 and 100% because each of the bootstrap samples must generate a proportion between 0 and 100%. Dr. Jarvis’ mistake appears to be that he estimated an average proportion and its variance from the ensemble of bootstrap estimates and then calculated the confidence interval using standard formulae (p. 10). The purpose of bootstrap estimation is to avoid such calculations, which can generate inaccurate confidence intervals. Although some of the bootstrap confidence intervals provided by Dr. Jarvis do not fall below 0% or surpass 100%, we guess that all of them are incorrectly calculated. Unfortunately, the incorrect confidence intervals are described by Dr. Jarvis (p. 12) as “mathematically and biologically implausible” and taken to “….betray the quantitative weaknesses in [Hertig *et al.*’s] data and analysis.” Indeed, they are “mathematically and biologically implausible” but the reason is that they were not correctly calculated. Whatever bearing a bootstrap analysis has on our understanding of the “precision” of Hertig *et al.*’s data and analyses remains to be seen.

 Dr. Jarvis’ central argument is that there is more ambiguity associated with estimates of fetal wastage in humans and that this ambiguity is not widely understood. Many of his concerns should be taken seriously. Nonetheless, his analysis is undermined by errors of analysis and overstatement. In the end, his estimate of fetal wastage from conception to birth is consistent with many of the previous estimates.

 Dr. Jarvis’ analysis is also undermined by an incorrect dismissal of data from embryos created via assisted reproductive technology (ART), which he refers to as *in vitro* fertilization (IVF). On page 11, he alludes to “…sub-optimal conditions for embryo culture…” and implies that somehow ART embryos are “different” in undefined ways from naturally-conceived embryos that negate their potential use in regard to estimating fetal wastage. This is an exercise in rhetoric, not a scientific argument. It is true that ART embryos are different from natural embryos in ways that could influence an estimate of fetal wastage. However, it is essential to note that they constitute the best available sample for insight into the “black box” of early pregnancy, despite the possible biases they may have that could distort our view into the black box. To this extent, it is best to assess what information they can provide about fetal wastage, rather than provide tenuous or irrelevant reasons as to why they are not useful.

 Dr. Jarvis mistakenly assumes (p. 11) that only ART embryos transferred into mothers would provide information about fetal wastage. In fact, as Dr. Jarvis notes, there are a number of reasons why transferred embryos are not representative of all embryos (e.g., conscious or unconscious quality biases, sex selection) and accordingly, this kind of sample could be misleading. That said, studies of such samples suggest that at least some aspects of their biology are identical to that of naturally-conceived embryos. For example, the sex ratio at birth for ART embryos is statistically identical with that of natural conceptions (Orzack *et al.*, 2015).

 More importantly, the entire ensemble of ART embryos (untransferred and transferred) provides information about fetal wastage. Almost all ART embryos undergoe testing for chromosomal abnormalities, such as aneuploidy. The consequences of aneuploidy are well-known – it results in almost certain death before birth. This is consistent with the fact that many spontaneous abortions are karyotypically abnormal (Boué, Boué, & Lazar, 1967, 1975; Jauniaux & Burton, 2005). To this extent, the frequency of such abnormalities provides strong circumstantial evidence as to the amount of fetal wastage. Orzack *et al. *(2015) investigated a sample of ART embryos whose karyotypes were assayed via FISH or CGH and reported that 84,881 out of 139,704 embryos contained at least one aneuploid chromosome. The implied percentage of fetal wastage (60.8%) is remarkably consistent with the central tendency of the many reports that Dr. Jarvis dismisses as unreliable, as well as with his own estimate. As noted, we need to be cautious about inferences from this sample but not avoid making them. There is no compelling reason to think that “suboptimal” conditions for embryo culture (if any) cause many chromosomal abnormalities, most of which very likely arise during meiosis (e.g., Hassold & Hunt, 2001; Hunt & Hassold, 2007; Jones, 2008; Nagaoka, Hassold, & Hunt, 2012). What deserves scrutiny are whether the frequency of chromosomal abnormalities is elevated by techniques for collecting eggs and/or because women providing them for use in ART are unrepresentative of all reproductive women. There are limited data that unstimulated and stimulated oocytes have similar frequencies of abnormality (Labarta *et al.*, 2010). Of course, women using ART are often older than many typical mothers. However, a high frequency of karyotypic abnormality is also observed among oocytes from young women (Baart *et al.*, 2006; Munné *et al.*, 2006). These concerns should continue to be investigated but they in no way imply that ART embryos cannot provide useful insights about early human development and fetal wastage, especially given the current lack and very likely continuing lack of a large sample of naturally-conceived human embryos.

 We see then a web of circumstantial evidence implying that there is a substantial amount of fetal wastage in humans. This insight arises from imperfect types of knowledge (as documented by Dr. Jarvis) but nonetheless, there is a signal consistent with the claim that approximately half or more of conceptions fail. More needs to be done to improve our understanding.

 The study of fetal wastage shares with the study of the human sex ratio during pregnancy the fact that many different kinds of scientists are involved and so, the associated balkanization has reduced the accountability that arises from a shared disciplinary perspective about the standards for the interpretation of data (Orzack, 2016; Orzack *et al.*, 2015). One cause and consequence of this division is the gray

 literature mentioned above. 

 What contributes to the continuing “life” of the gray literature? Science abhors a vacuum and claims about high fetal wastage in humans have been repeated often in a way that the connection with assumptions and data have gotten obscured or lost. Some claims date well before there was any means by which early mortality could be assessed (Mall, 1917; Meyer, 1920; Pearson, 1897). Pearson clearly acknowledged the lack of direct evidence but such caveats get lost especially in medicine in which attention to standards of evidence, recognition of the assumptions needed to connect data with conclusions, and awareness of needed statistical techniques have been less as compared to in biological research. These deficiencies have diminished as medical training has incorporated more scientific training but have not disappeared. Nonetheless, during medical training the “inhalation” of facts is important. It is one reason as to why many believe that fetal wastage is high, despite having little or no familiarity with the available data along with the ins and outs of their analysis and interpretation.

 In this context, care is needed when assessing the nature of claims about fetal wastage. This can be illustrated by considering Dr. Jarvis’ claim (p. 8):

*….it is clear that estimates for total embryonic loss of 90% (Opitz, 2002), 85% (Braude & Johnson, 1990), 83% (Harris, 2003), 80– 85% (Johnson & Everitt, 2000; Vitzthum, Spielvogel, Thornburg, & West, 2006), 78% (Roberts & Lowe, 1975), 76% (Boklage, 1990; Drife, 1983) and 70% (Chard, 1991; Ford & Schust, 2009; Loke & King, 1995; Macklon, Geraedts, & Fauser, 2002; McCoy et al., 2015) are excessive.*

 (We have replaced number citations with author citations). Several of these claims are in medical textbooks and are akin to newspaper articles, i.e., they are reports on prior research as opposed to being independent estimates. Even then the nature of the evidence can go unmentioned. For example, in their text book Johnson & Everitt (2000) include no evidence or citations in which to find evidence

 underlying their estimate. Of the claims in the primary literature, we again see a lack of independent evidence in as much as someone else’s estimate is reported. For example, Chard (1991); Drife (1983); Vitzthum *et al. *(2006) merely present Roberts & Lowe's (1975) estimate. A few claims present their own evidence. For example, Harris (2003) contains this passage (p. 362): 

*We now know that for every successful pregnancy that results in a live birth many, perhaps as many as five early embryos will be lost or will “miscarry”….*

 and accompanying footnote (p. 371):

*Robert Winston gave the figure of five embryos for every live birth some years ago in a personal communication. Anecdotal evidence to me from a number of sources confirms this high figure, but the literature is rather more conservative, making more probable a figure of three embryos lost for every live birth. See: Boklage CE. Survival probability of human conceptions from fertilization to term. International Journal of Fertility 1990;35(2)75–94. See also: Leridon H. Human Fertility: The Basic Components. Chicago: University of Chicago Press; 1977. Again, in a recent personal communication, Henri Leridon confirmed that a figure of three lost embryos for every live birth is a reasonable conservative figure.*

 This is clearly a heuristic estimate! The point is that there is less of a monolithic ensemble of flawed estimates that need to be debunked than one might imagine given Dr. Jarvis’ passage. In any case, there is nothing inherently problematic about the citations just described. Indeed, it would be preferable if attributions were better and speculation was better highlighted as such. Nonetheless, such estimates should be used with caution but not discarded, given the substantial difficulties associated with the estimation of fetal wastage in humans.

 An ideal future investigation of fetal wastage is easy to imagine: daily assessment of EPF and hCG for a cohort of women attempting to get pregnant. Easier said than done! Consider what such a study would require: a reliable assay for EPF, the enrollment of thousands of women, collection of and accurate assessment of thousands of samples, and more. Perhaps these technical and logistical barriers can be overcome soon. In the meantime, we can recognize that there is strong circumstantial evidence that human fetal wastage is likely between 50 and 75%. At the same time, we can recognize along with Dr. Jarvis that this conclusion lacks definitive proof and that additional investigations and scrutiny are needed.

We have read this submission. We believe that we have an appropriate level of expertise to confirm that it is of an acceptable scientific standard, however we have significant reservations, as outlined above.

## References

[1] AustinC. R.: Pregnancy losses and birth defects. *Reproduction in Mammals 2: Embryonic and Fetal Development*.1972;134-153

[2] BaartEBMartiniEvan den BergIMacklonNSGaljaardRJFauserBCVan OpstalD: Preimplantation genetic screening reveals a high incidence of aneuploidy and mosaicism in embryos from young women undergoing IVF. *Hum Reprod*.2006;21(1) : 10.1093/humrep/dei291 223-33 10.1093/humrep/dei291 16155075

[3] BoklageCE: Survival probability of human conceptions from fertilization to term. *Int J Fertil*.35(2) :75, 79-80, 81 1970983

[4] BouéJGBouéALazarP: [Chromosome aberrations in abortions]. *Ann Genet*.1967;10(4) :179-87 5301690

[5] BouéJBou ALazarP: Retrospective and prospective epidemiological studies of 1500 karyotyped spontaneous human abortions. *Teratology*.1975;12(1) : 10.1002/tera.1420120103 11-26 10.1002/tera.1420120103 1162621

[6] BrambellF.W.R: Intra-Uterine Mortality of the Wild Rabbit, Oryctolagus cuniculus (L.). *Proceedings of the Royal Society of London. Series B - Biological Sciences*.1942;130(861) :462-479 Reference source

[7] BRAMBELLFW: Prenatal mortality in mammals. *Biol Rev Camb Philos Soc*.1948;23(4) :370-407 1810435810.1111/j.1469-185x.1948.tb00565.x

[8] BraudeP. RJohnsonM. H.: The embryo in contemporary medical science. *The Human Embryo: Aristotle and the Arabic and European Traditions*.1990;208-221

[9] CasidaL. E.: Fertilization failure and embryonic death in domestic animals. *Pregnancy Wastage. Thomas, Springfield, Ill*.1953;27-37

[10] ChardT: Frequency of implantation and early pregnancy loss in natural cycles. *Baillieres Clin Obstet Gynaecol*.1991;5(1) :179-89 185533910.1016/s0950-3552(05)80077-x

[11] ClarkeFM: Controversies in assisted reproduction and genetics. Does. *J Assist Reprod Genet*.1997;14(9) :489-91 940186210.1023/A:1021110906666PMC3454847

[12] DrifeJ. O.: What proportion of pregnancies are spontaneously aborted?. *British Medical Journal, 286*.1983;294

[13] EfronB.: Better bootstrap confidence intervals. *Journal of the American statistical Association*.1987;82(397) :171-185 Reference source

[14] EfronB.TibshiraniR. J.: Bootstrap methods for standard errors, confidence intervals, and other measures of statistical accuracy. *Statistical Science*.1986;1(1) :54-75 Reference source

[15] FanXGZhengZQ: A study of early pregnancy factor activity in preimplantation. *Am J Reprod Immunol*.1997;37(5) :359-64 9196793

[16] FirstN. L.EyestoneW. H.: Reproductive efficiency in domestic animals. *Annals of the New York Academy of Science*.1988;541(1) :697-705 Reference source 10.1111/j.1749-6632.1988.tb22307.x3195946

[17] FordHBSchustDJ: Recurrent pregnancy loss: etiology, diagnosis, and therapy. *Rev Obstet Gynecol*.2009;2(2) :76-83 19609401PMC2709325

[18] FRENCHFEBIERMANJM: Probabilities of fetal mortality. *Public Health Rep*.1962;77:835-47 13959550PMC1915016

[19] HarrisJ: Stem cells, sex, and procreation. *Camb Q Healthc Ethics*.2003;12(4) :353-71 1461936810.1017/s096318010312405x

[20] HassoldTHuntP: To err (meiotically) is human: the genesis of human aneuploidy. *Nat Rev Genet*.2001;2(4) : 10.1038/35066065 280-91 10.1038/35066065 11283700

[21] HertigA. T.: The overall problem in man. *In Comparative Aspects ofReproductive Failure Springer Berlin Heidelberg*.1967;11-41 Reference source

[22] HertigATRockJ: Searching for early fertilized human ova. *Gynecol Invest*.1973;4(3) :121-39 459397310.1159/000301716

[23] HERTIGATROCKJADAMSECMENKINMC: Thirty-four fertilized human ova, good, bad and indifferent, recovered from 210 women of known fertility; a study of biologic wastage in early human pregnancy. *Pediatrics*.1959;23(1 Part 2) :202-11 13613882

[24] HuntPAHassoldTJ: Human female meiosis: what makes a good egg go bad?. *Trends Genet*.2008;24(2) : 10.1016/j.tig.2007.11.010 86-93 10.1016/j.tig.2007.11.010 18192063

[25] JauniauxEBurtonGJ: Pathophysiology of histological changes in early pregnancy loss. *Placenta*.26(2-3) : 10.1016/j.placenta.2004.05.011 114-23 10.1016/j.placenta.2004.05.011 15708112

[26] JohnsonM. H.EverittB. J.: *Essential Reproduction (5th ed.)*.2000;

[27] JonesKT: Meiosis in oocytes: predisposition to aneuploidy and its increased incidence with age. *Hum Reprod Update*.14(2) : 10.1093/humupd/dmm043 143-58 10.1093/humupd/dmm043 18084010

[28] LabartaE.BoschE.AlamaP.RubioC.RemohíJ.PellicerA.: Ovarian stimulation does not increase embryo aneuploidy rates in young normo-ovulatory women. *Human Reproduction*.2010;25:I62-I63

[29] LasleyE. L.: Ovulation, prenatal mortality and litter size in swine. *Journal of Animal Science*.1957;2(16) :335-340 Reference source

[30] LéridonH: Human fertility: The Basic Components. *Chicago: University of Chicago Press.*.1977;

[31] LokeY. W.KingA.: Human Implantation: Cell Biology and immunology. *Cambridge:Cambridge University Press.*.1995;

[32] MacklonNSGeraedtsJPFauserBC: Conception to ongoing pregnancy: the 'black box' of early pregnancy loss. *Hum Reprod Update*.8(4) :333-43 1220646810.1093/humupd/8.4.333

[33] MallF. P.: Note on abortions with letters from the Health Commissioner of Baltimore and from the Chief of the Bureau of Vital Statistics of Maryland regarding registration andshipment of embryos to the Carnegie Laboratory of Embryology at the Johns Hopkins Medical. Baltimore. 1917;

[34] McCoyRCDemkoZRyanABanjevicMHillMSigurjonssonSRabinowitzMFraserHBPetrovDA: Common variants spanning PLK4 are associated with mitotic-origin aneuploidy in human embryos. *Science*.2015;348(6231) : 10.1126/science.aaa3337 235-8 10.1126/science.aaa3337 25859044PMC5519344

[35] MeyerAW: THE PRACTITIONER'S CONTRIBUTION TO EMBRYOLOGY. *Cal State J Med*.1920;18(2) :44-6 18738102PMC1594116

[36] MortonH.RolfeB. E.CavanaghA. C.: Early pregnancy factor. *Seminars in Reproductive Endocrinology*.1992;10(2) :72-82

[37] MunnéSAryJZouvesCEscuderoTBarnesFCiniogluCAryBCohenJ: Wide range of chromosome abnormalities in the embryos of young egg donors. *Reprod Biomed Online*.2006;12(3) :340-6 1656932410.1016/s1472-6483(10)61007-3

[38] NagaokaSIHassoldTJHuntPA: Human aneuploidy: mechanisms and new insights into an age-old problem. *Nat Rev Genet*.2012;13(7) : 10.1038/nrg3245 493-504 10.1038/nrg3245 22705668PMC3551553

[39] NepomnaschyPAWeinbergCRWilcoxAJBairdDD: Urinary hCG patterns during the week following implantation. *Hum Reprod*.2008;23(2) : 10.1093/humrep/dem397 271-7 10.1093/humrep/dem397 18083748PMC5330618

[40] OpitzJ. M.: Human development - the long and the short of it. In E. J. Furton.2002;

[41] OrzackS. H.: Old and new ideas about the human sex ratio. *Significance*.2016;13(1) : 10.1111/j.1740-9713.2016.00879.x 24-27 10.1111/j.1740-9713.2016.00879.x

[42] OrzackSHStubblefieldJWAkmaevVRCollsPMunnéSSchollTSteinsaltzDZuckermanJE: The human sex ratio from conception to birth. *Proc Natl Acad Sci U S A*.2015;112(16) : 10.1073/pnas.1416546112 E2102-11 10.1073/pnas.1416546112 25825766PMC4413259

[43] PearsonK.: The Chances of Death and Other Studies in Evolution. *CUP Archive*.1897;1:

[44] RobertsC. J.LoweC. R.: Where have all the conceptions gone?. *Problems of Birth Defects*.1975;148-150

[45] RolfeBE: Detection of fetal wastage. *Fertil Steril*.1982;37(5) :655-60 617647410.1016/s0015-0282(16)46278-3

[46] ShahaniSKMonizCChitlangeSMeherjiP: Early pregnancy factor (EPF) as a marker for the diagnosis of subclinical embryonic loss. *Exp Clin Endocrinol*.1992;99(3) : 10.1055/s-0029-1211152 123-8 10.1055/s-0029-1211152 1526259

[47] ShahaniSKMonizCLGokralJSMeherjiPK: Early pregnancy factor (EPF) as a marker for detecting subclinical embryonic loss in clomiphene citrate-treated women. *Am J Reprod Immunol*.1995;33(5) :350-3 757611610.1111/j.1600-0897.1995.tb00903.x

[48] SmartYCFraserISRobertsTKClancyRLCrippsAW: Fertilization and early pregnancy loss in healthy women attempting conception. *Clin Reprod Fertil*.1982;1(3) :177-84 6196101

[49] VitzthumVJSpielvogelHThornburgJWestB: A prospective study of early pregnancy loss in humans. *Fertil Steril*.2006;86(2) : 10.1016/j.fertnstert.2006.01.021 373-9 10.1016/j.fertnstert.2006.01.021 16806213

[50] WangXChenCWangLChenDGuangWFrenchJ: Conception, early pregnancy loss, and time to clinical pregnancy: a population-based prospective study. *Fertil Steril*.2003;79(3) :577-84 1262044310.1016/s0015-0282(02)04694-0

[51] WilcoxAJBairdDDWeinbergCR: Time of implantation of the conceptus and loss of pregnancy. *N Engl J Med*.1999;340(23) : 10.1056/NEJM199906103402304 1796-9 10.1056/NEJM199906103402304 10362823

[52] WilcoxAJWeinbergCRO'ConnorJFBairdDDSchlattererJPCanfieldREArmstrongEGNisulaBC: Incidence of early loss of pregnancy. *N Engl J Med*.1988;319(4) : 10.1056/NEJM198807283190401 189-94 10.1056/NEJM198807283190401 3393170

[53] ZinamanMJCleggEDBrownCCO'ConnorJSelevanSG: Estimates of human fertility and pregnancy loss. *Fertil Steril*.1996;65(3) :503-9 8774277

# Response

**Published:** 2017-05-31

**Authors:** Gavin Jarvis

**Affiliations:** University of Cambridge, UK

I would like to thank Professor Orzack and Professor Zuckerman for their extensive review. I have amended the article in light of their remarks, and hope they consider it improved as a result.

**Introduction**

The purpose of my article is to evaluate available data that contribute to our quantitative understanding of natural human embryo mortality. The body of relevant data is small, as noted by the reviewers, although I have attempted to identify all of it. I deliberately avoided IVF/ART data since there is so much, and it is not obvious how such data illuminate natural circumstances (I comment further on this below). My comments on IVF/ART data are therefore confined to the Discussion. 

Orzack & Zuckerman repeatedly refer to my estimates of 10-40% preimplantation loss and 40-60% total embryo loss, which are important benchmarks for my article. They are critical of these, although they do not seem to appreciate where they come from. Contrary to what they imply (“On the basis of his review of this evidence…”), they do not arise from analyses described in this article. Rather, they are from an analysis described in a previous article in *F1000Research* ^1^. I have amended the article to clarify this point. Concerns with the validity of these estimates should focus on that analysis, which is not listed among their 53 references.

In their review, the reviewers are ambiguous (one might say ‘gray’) in their use of quotation marks and appear to ascribe to me things I did not write. For example, I do not use the phrase “great deal”. Thus, for the sake of clarity, and to separate literary emphasis from quotation, I will follow the convention employed by GEM Anscombe, who coined a useful phrase ^2^, to distinguish between ‘scare quotes’ and “quotations”.

I address points raised in the review, approximately in the order in which they appear.

**1. Roberts & Lowe**

Orzack & Zuckerman state that I calculate 95% confidence intervals. This is incorrect. The range [37-90%] is not a confidence interval, I do not refer to it as such, and nor can it be, since there are no data. As described in the article, it is the range within which 95% of simulated estimates fall, based on Roberts & Lowe’s speculative values and other assumptions.

The reviewers suggest that my analysis lacks “practical quantitative value”. I agree. This is the point and I am glad they have recognised it, if not entirely appreciated its significance. My analysis has “no practical quantitative value” *for estimating the number of conceptions that are lost*. As I explicitly point out, the sole purpose of the sensitivity analyses is to show that modest changes in the speculative estimates used by Roberts & Lowe may result in any biologically plausible value for embryo loss. 

That my simulated estimate of 76.5% is close to Roberts & Lowe’s 78% is not telling since it uses their original speculative values. On the contrary, it would be telling (of something) if they were not close. I simply added variance to the speculative values. I comment on the nature of this variance/covariance in my response to Reviewer 2. Thus, my analysis does not validate Roberts & Lowe, it exposes its quantitative futility.

Gray Literature is “documentary material which is not commercially published or publicly available, such as technical reports or internal business documents.” ^3^ *The Lancet *is not ‘Gray Literature’. I comment further on this below. Contrary to the reviewers’ suggestion, we are not completely “ignorant of the training of Drs. Roberts & Lowe” or unaware of their experience in “research practice and data analysis”. Charles Ronald Lowe was the more senior of the two. He was 63 years old and Professor of Social and Occupational Medicine at the University of Wales College of Medicine when *The Lancet* article was published. He “contributed much to the growth of academic public health and the teaching of epidemiology and statistics.” ^4^

I do not describe their work as “useless” – if intended as a quote, then it is a misquote. I describe it as having “no practical quantitative value”. These are carefully chosen words. (I have edited the equivalent phrase in the Abstract to match the full text.) The critique offered by the reviewers and their description of the paper as heuristic support this view. Nevertheless, I have added a statement that, as a model for highlighting factors that influence fecundity, the Roberts & Lowe analysis has some value. 

In all fairness, on four separate occasions, I describe the analysis of Roberts & Lowe as a “hypothesis”, i.e., the banner under which it was originally published in *The Lancet*. Indeed, they describe their arithmetic as “speculative”; however, they also describe their estimate as “conservative”, implying that the true result may be even higher than 78%. My critique would be less germane had their hypothesis not been cited so widely (“more than 300 times”, as helpfully pointed out by the reviewers). I suggest that it is not I, but those who enthusiastically cite it ^5^ who treat it as “something that it isn’t”.

**2. Life Tables of Intrauterine Mortality**

I do not consider Leridon’s chapter ^6^ a “casual exercise” or “superficial”. On the contrary, it is a well-reasoned attempt to answer a challenging biological question. I have included a tribute in my article to Leridon’s review. I hope this prevents readers from gaining such false impressions.

I agree with the reviewers that Leridon’s 63% is close to my 40-60%. However, Roberts & Lowe’s 78% is not, as they imply.

A critique of Leridon’s life-table is not a critique of Leridon at all, but of French & Bierman ^7^ and Hertig ^8^. I discuss briefly why French & Bierman may be an overestimate and, in detail, how Hertig’s analysis is flawed. Leridon’s account has been widely cited, especially by those describing embryo loss at the earliest stage. I hope readers will find it useful to know how Leridon’s values are derived.

**3. hCG studies of early pregnancy loss**

The Edmonds (1982) estimate of approximately 60% loss ^9^ is the highest I report and, for reasons discussed in the article and mentioned by others ^10^, is likely to be an over-estimate. Nevertheless, years after the more credible Wilcox (1988) ^10^ study was published, Edmonds is still widely cited to justify high levels of embryo wastage. For example, Hyde & Schust (2015) ^11^ cite both Edmonds and Wilcox to support their claim that “Approximately 70% of human conceptions fail to achieve viability, with almost 50% of all pregnancies ending in miscarriage before the clinical recognition of a missed period…” By showing Edmonds’ results in context, I hope this kind of overstatement can be avoided.

My conclusion of one third loss is based on the average of the eight listed studies from Wilcox to the present day (unweighted average = 31.9%). I have edited the paper to make this clear. I also discuss why the estimates prior to Wilcox are less reliable and cite several studies that make similar observations.

As already noted, my 40-60% estimate is from a previously analysis ^1^ and is not a combination of the values (31.3; 35.7; 31.3) highlighted by the reviewers. My rationale for using a 90% implantation (and fertilisation) efficiency is found in that analysis ^1^. 

My conclusion regarding the validity of Boklage’s analysis of embryo mortality ^12^ is not “unsubstantiated”. Indeed, the reviewers mention a key point of substance: namely, that Boklage’s value of 28.7% misinterprets the biology. Boklage uses this as measure of embryo mortality, whereas it is a fecundability. If fecundabilities are analysed as embryo mortalities, surely this casts doubt on the validity of conclusions regarding embryo mortality.

I cannot comment on Boklage’s statistical methodology (i.e., use of MLE, LRTs or AIC values) since he reports no such detail. However, I thank the reviewers for highlighting the lack of clarity in Boklage’s analysis. 

Contrary to the claim of the reviewers, I refer to Boklage’s estimate of 76% loss from conception (fertilisation) to birth on three occasions. This 76% estimate is consistent with Roberts & Lowe’s value. It is somewhat higher than Leridon’s (whose life table is inexplicably omitted from the Boklage analysis). It is clearly not consistent with my 40-60% estimate ^1^. 

**4. Hertig’s data and analysis**

Regarding Hertig’s conclusion, Orzack & Zuckerman “believe that it has been well recognized that it is ‘impressionistic’ as opposed to something that has a solid quantitative underpinning”. I agree that Hertig’s conclusion does not have a “solid quantitative underpinning”; however, it is precisely the quantitative underpinning of Leridon’s life table and other claims about early natural embryo mortality. This is a key point of my article. It is not clear what the reviewers mean by ‘impressionistic’ ^13^: some authors seem to offer an ‘unimpressionistic’ account of Hertig. For example, in the widely-cited ‘Black Box’ review ^14^, Macklon *et al*. write regarding Hertig’s study: “…the high rate of early pregnancy loss before the time of the first missed period was thus clearly demonstrated…” Other less widely-cited articles ^15^ do address the design and analytical shortcomings. 

Pointing out shortcomings in studies is what scientists (and reviewers) are meant to do. Thus, I agree with the reviewers that they are “worth mentioning”. Furthermore, by pointing out that Hertig’s subjects were of proven fertility, had gynaecological problems and may have had suboptimal reproductive function, I am placing Hertig’s study “in proper context”. This is not “un-useful”. Nevertheless, I have edited this section, to accommodate these reviewers’ scepticism with the more positive view of others ^16^. I hope I have struck an acceptable balance. 

Orzack & Zuckerman appear to have concerns with well-established statistical techniques, referring to my “so-called ‘bootstrap’ samples”. I agree that bootstrapping is “not a ‘cure’ for small sample size”, but I do not claim that it is. Bootstrapping can provide estimates of precision when it is not possible to calculate these analytically. As with all analyses, outputs require appropriate interpretation.

The reviewers state that the “analyses of the bootstrap results are incorrect” because some of the confidence intervals lie outside the range 0-100%. I am aware that this is impossible (for a probability) as I explicitly point out. Such outputs do indicate a serious flaw in the analysis, which is as follows: Hertig ignores 47 of his 107 cases. These cases are included in my bootstrap. The reader may consider whether ignoring 44% of the data is reasonable and the extent to which by doing so Hertig has generated biased estimates of the probabilities he calculates. Kline *et al*. (1989) make a similar point: “The missing data are sufficient to engender an entirely different result” ^15^. The bootstrap therefore illustrates the extent to which Hertig’s estimates are biased by ignoring his own data. There are other reasons to doubt the precision of his conclusions and the representative nature of the subset of data upon which he relies so heavily – these are described in the article. 

The bootstrap pseudo-datasets are available for scrutiny (Dataset 4). Thus, if there are any flaws in my reasoning or bootstrap, the reviewers may point these out. I used the percentile method (to which they refer) to calculate the 95% CIs and I have edited the text to clarify this. I do not believe there are any flaws in my bootstrap.

**IVF/ART data**

There is a wealth of data from IVF/ART studies and I have only mentioned a tiny proportion of this. Orzack & Zuckerman and a previous reviewer ^17^ suggest that such data could contribute to a quantitative understanding of the *in vivo* situation. In the broadest sense, this is of course true. However, there are difficulties in extrapolating from *in vitro* to *in vivo* circumstances. I am not alone in pointing this out ^14^,  and I have illustrated some of these difficulties in the Discussion.

My description of “sub-optimal conditions for embryo culture” is drawn from two papers:

1. Bolton & Braude (1987) ^19^: “Optimal culture conditions for human embryos have yet to be defined” and “suboptimal culture conditions are undoubtedly responsible for a proportion of this embryonic failure”. 
2. Bolton *et al*. (2015) ^20^: “Embryo culture conditions *in vitro* are likely to be suboptimal compared to those *in vivo*.”

Is this just rhetoric or a reasonable consideration?

Describing *in vitro* data as the “best available” is a weak claim in the absence of equivalent natural *in vivo* data. The extent to which *in vitro* embryos are representative of *in vivo* embryos is precisely the point in question. Is there really numerical consistency between natural and IVF/ART embryos? There may be consistency in sex ratios ^21^, but does that extend to aneuploidy rates, mosaicism, epigenetic defects, implantation potential, spontaneous abortion rates, etc? These are big questions and this article is not the place to answer them. However, if 70% loss ^14^ is the natural benchmark by which IVF/ART embryos are judged to be equivalent to natural embryos ^22^, but the true rate of natural loss lies in the range 40-60%, this therefore casts doubt on the judgement that IVF/ART and natural embryos are equivalent. Furthermore, the suggestion that IVF/ART and natural embryos may be different is neither radical, novel, nor strong ^23^. However, the real reason I do not consider IVF/ART embryo data is that the article is a critique of data from natural circumstances. Comparison of natural and IVF/ART embryos is a project for the future.

The reviewers refer to my “tenuous or irrelevant reasons” why ART embryos are not useful for quantifying early embryo mortality, yet they provide the perfect reason themselves: “it is true that ART embryos are different from natural embryos in ways that could influence an estimate of fetal wastage” ^24^. Nevertheless, I do discuss circumstances in which different ART interventions (e.g., observation of *in vitro* fertilisation *per se*; retrieval of embryos following timed artificial insemination, as well as AID/IVF success rates) may cast light on embryonic/fetal wastage. 

Orzack & Zuckerman extrapolate from 84,881 aneuploidies among 139,704 IVF/ART embryos ^21^ to an “implied percentage of fetal wastage” of 60.8%. They state that this is the “central tendency” of “many reports” that I dismiss as unreliable. Of course, if this were true, then the observation would add little to what was already known. It is not clear which are the “many reports”.

Let us consider the hypothesis that *in vitro* aneuploidy predicts natural total fetal wastage. Firstly, “The only well-established epidemiological facts about EPL { *early pregnancy loss*} are that about 50-60% of cases are associated with a chromosomal defect of the conceptus” ^25^ suggesting that euploid embryos may also fail. Secondly, “FISH may overestimate the incidence of aneuploidy” ^21,26^ suggesting a proportion of apparently aneuploid embryos may not fail. Furthermore, aneuploidy may not developmentally compromise embryos ^27^; estimates of IVF/ART embryo aneuploidy/mosaicism vary considerably ^28^; mosaic embryos can self-correct ^29^; aneuploidy in trophoblast/placental cells may be less developmentally problematic ^23^ – who knows, it may even be advantageous!

The point is simple. There are too many undefined variables associated with IVF/ART embryos to shed more than the faintest light on the question of natural embryo survival. I have included a brief discussion of some of these issues and edited the penultimate paragraph to be more circumspect by replacing an “are” with a “may be”. I hope this meets with the reviewers’ approval.

**Gray Literature**

On several occasions, the reviewers refer to Gray Literature. They offer a revealing account and speculate on its continuing ‘life’. 

Gray Literature has been defined as follows: “That which is produced on all levels of government, academics, business and industry in print and electronic formats, but which is not controlled by commercial publishers.” ^30,31^

The list of references reproduced by the reviewers, starting with Opitz, 2002 and ending with McCoy *et al*. 2015 are all from academic books, journals, or text books. They are all published by commercial publishers. They were all written (with one exception) by medical practitioners or scientists, many of whom are experts in reproductive biology. The one exception (Harris, 2003) is a moral philosopher; however, the reviewers usefully point out that his estimate comes from a well-known and eminent reproductive biologist. 

None of this is Gray Literature. *Human Reproduction Update*, *Fertility & Sterility* & *PLOS Genetics* are reputable academic journals. Many of these articles will have been peer-reviewed. Even pieces “akin to newspaper articles” (the Drife (1983) *BMJ* piece could be described as such and was probably not peer-reviewed ^32^) are subject to editorial control, and an expectation of academic professionalism is surely reasonable from such experts.

The reviewers state that it would “be preferable if attributions were better and speculation was better highlighted”. I agree. Yet they highlight my 'so-called' “errors of analysis” and “overstatement” whilst passing over errors and overstatement in these citations as “nothing inherently problematic”.

What Orzack & Zuckerman describe and defend is not Gray Literature, but ‘Gray Scholarship’.

**Heuristics**

A heuristic estimate may be based on simplified quantitative criteria, educated guesswork, rules of thumb, common sense, past experience, etc. Despite their utility, in the absence of evidence heuristic estimates may become biased. Faced with inconsistent estimates, on the one hand, those that are heuristic or based on circumstantial evidence, and on the other, those based on well-defined analysis of relevant data, surely an appropriate scientific response is to favour the latter and re-evaluate the former.

A further problem with heuristic estimates is that the process for deriving them is not always transparent. For example, it is not obvious how Orzack & Zuckerman use the “web of circumstantial evidence” to which they refer to conclude that “human fetal wastage is likely between 50 and 75%”. There is something ‘gray’ about this. My estimates of 10-40% preimplantation loss and 40-60% total loss are partly evidence-based and partly heuristic. They may be imperfect, and no doubt will not be the last word on the matter, but it is at least clear how they were derived ^1^.

**Conclusion**

Orzack & Zuckerman often repeat the point that my estimates are consistent with previously published values. In some cases they are, and I have drawn more attention to the fine chapter by Kline *et al*. (1989) ^15^  who conclude that “perhaps half of all conceptions are lost before birth” ^33^. However in other cases, reported values are clearly not consistent with my estimates. I have used 70% total embryo loss as a threshold, at and above which I describe estimates as exaggerated. This is based on my previous analysis ^1^ and thus my claim rests heavily, although not solely, on its credibility. There are other reasons to cast doubt on these high values, but these are for another time. I have modified the conclusion of my article to highlight that while precision may be elusive, exaggeration can be avoided.

“Nature abhors a vacuum”, so the proverb says, but how science, or more properly scientists, should fill it is another matter entirely. Recognising and quantifying limits of knowledge is an essential part of a credible scientific process. As a philosopher once wrote: “Wovon man nicht sprechen kann, darüber muss man schweigen” ^34^.

**References:**

1. Jarvis GE: **Estimating limits for natural human embryo mortality [version 2; referees: 2 approved]** **.** *F1000Res.* 2016, **5**:2083 (doi: 10.12688/f1000research.9479.2).
2. Anscombe GEM: **Aristotle and the Sea Battle.** *Mind*, 1956; **65**(257):1-15.
3. Oxford English Dictionary ( http://www.oed.com/).
4. Roberts CJ: **Obituary: C R Lowe.** *Brit Med J*. 1994; **308**(6921): 129.
5. “It is still difficult to better the original calculations of Roberts & Lowe (1975)” from: Chard T: **Frequency of implantation and early pregnancy loss in natural cycles**. *Baillieres Clin Obstet Gynaecol*. 1991; **5**(1): 179-89.
6. Leridon H: **Intrauterine Mortality**. *Human Fertility: The Basic Components*. Chicago: The University of Chicago Press; 1977; 48-81.
7. French FE, Bierman JM: **Probabilities of fetal mortality**. *Public Health Rep*. 1962; **77**(10): 835-47.
8. Hertig AT: **The Overall Problem in Man.** In: Benirschke K, editor. *Comparative Aspects of Reproductive Failure*. An International Conference at Dartmouth Medical School. Berlin: Springer Verlag; 1967; 11-41.
9. Edmonds DK, Lindsay KS, Miller JF, et al.: **Early embryonic mortality in women.** *Fertil Steril*. 1982; **38**(4): 447-53.
10. Wilcox AJ, Weinberg CR, O'Connor JF, et al.: **Incidence of early loss of pregnancy.** *N Engl J Med*. 1988; **319**(4): 189-94.
11. Hyde KJ, Schust DJ: **Genetic considerations in recurrent pregnancy loss**. *Cold Spring Harb Perspect Med*. 2015; **5**: a023119.
12. Boklage CE: **Survival probability of human conceptions from fertilization to term**. *Int J Fertil*. 1990; **35**(2): 75, 79-80, 81-94.
13. According to Wikipedia, scare quotes too often serve to confuse rather than clarify ( https://en.wikipedia.org/wiki/Scare_quotes#Criticism).
14. Macklon NS, Geraedts JP, Fauser BC: **Conception to ongoing pregnancy: the ‘black box’ of early pregnancy loss**. *Hum Reprod Update*. 2002; **8**(4): 333-43.
15. Kline J, Stein Z, Susser M: **Conception and Reproductive Loss: Probabilities.** *Conception to Birth. Epidemiology of Prenatal Development*. New York: OUP; 1989; 43-68.
16. Saunders P,  Gibson DA. **Referee Report For:  Early embryo mortality in natural human reproduction: What the data say [version 1; referees: 1 approved, 2 approved with reservations].** *F1000Research* 2016, **5**:2765 (doi: 10.5256/f1000research.9616.r19546)
17. Trounson AO. **Referee Report For:  Estimating limits for natural human embryo mortality [version 2; referees: 2 approved]**. *F1000Research* 2016, **5**:2083 (doi: 10.5256/f1000research.10209.r16765).
18. Benagiano G, Farris M, Grudzinskas G: **Fate of fertilized human oocytes**. *Reprod Biomed Online*. 2010; **21**(6): 732-41.
19. Bolton VN, Braude PR: **Development of the human preimplantation embryo in vitro**. *Curr Top Dev Biol*. 1987; **23**: 93-114.
20. Bolton VN, Leary C, Harbottle S, et al.: **How should we choose the ‘best’ embryo? A commentary on behalf of the British Fertility Society and the Association of Clinical Embryologists**. *Hum Fertil (Camb)*. 2015; **18**(3): 156-64.
21. Orzack SH, Stubblefield JW, Akmaev VR, et al.: **The human sex ratio from conception to birth**. *Proc Natl Acad Sci USA*. 2015; **112**(16): E2102-11.
22. Niakan KK, Han J, Pedersen RA, et al.: **Human pre-implantation embryo development**. *Development*. 2012; **139**(5): 829-41.
23. Ledbetter DH: **Chaos in the embryo**. *Nat Med*. 2008; 14(5): 490-1.
24. Zuckerman JE,  Orzack SH. **Referee Report For:  Early embryo mortality in natural human reproduction: What the data say [version 1; referees: 1 approved, 2 approved with reservations]**. *F1000Research*. 2016, **5**:2765 (doi: 10.5256/f1000research.9616.r22945).
25. Jauniaux E,  Burton GJ. **Pathophysiology of histological changes in early pregnancy loss**. *Placenta*, 2005; **26**: 114-23.
26. Treff NR, Levy B, Su J, et al.: **SNP micro-array based 24 chromosome aneuploidy screening is significantly more consistent than FISH**. *Mol Hum Rep*. 2010; **16**(8): 583-9.
27. Bolton H, Graham SJL, Van der Aa N, et al.: **Mouse model of chromosome mosaicism reveals lineage-specific depletion of aneuploid cells and normal developmental potential**. *Nat Commun*. 2016; **7**: 11165.
28. Vanneste E, Voet T, Le Caignec C, et al.: **Chromosome instability is common in human cleavage-stage embryos**. *Nat Med*. 2009; **15**(5): 577-83.
29. Munne S, Velilla E, Colls P, et al.: **Self-correction of chromosomally abnormal embryos in culture and implications for stem cell production**. *Fertil Steril*. 2005; **84**(5): 1328-34.
30. The Grey Literature Report ( http://www.greylit.org/about).
31. See also: Wikipedia ( https://en.wikipedia.org/wiki/Grey_literature); GreyNet International 1992-2017 ( http://www.greynet.org/home/aboutgreynet.html); California State University, Long Beach Libraries ( http://csulb.libguides.com/graylit).
32. Personal Communication from *Brit Med J*, 18 ^th^ April 2016.
33. Kline J, Stein Z, Susser M: **Developmental Abnormalities: I. Measuring Frequencies.** *Conception to Birth. Epidemiology of Prenatal Development*. New York: OUP; 1989; 69-80.
34. Wittgenstein L: **Tractatus 7** *Tractatus Logico-Philosophicus*. New York: Harcourt, Brace & Co, Inc.; 1922 (translated as: *Whereof one cannot speak, thereof one must be silent*).

# Peer review: Referee response for version 1

**DOI:** 10.5256/f1000research.9616.r19502

**Published:** 2017-02-09

**Authors:** Jack Wilkinson

**Affiliations:** 1 Centre for Biostatistics, School of Health Sciences, Faculty of Biology, Medicine and Health, Manchester Academic Health Science Centre (MAHSC), University of Manchester, Manchester, UK

Thanks for the opportunity to review this high-quality manuscript. Peer review can be a chore, but this was a pleasure to read.

 I will state that my training is in statistics and research methodology. Although much of my work is in the field of fertility, I have no clinical expertise and no familiarity with the literature discussed in this review. Any comments I make are from the point of view of the statistician and, with respect to the subject-matter, the layperson.

 I am unable to comment on whether or not the body of evidence discussed in the review is comprehensive. However, the critical appraisal of these studies is conducted to a high standard, with a strong command of quantitative research methods on display. I can’t fault it. The reader is left in no doubt as to the considerable limitations (many of which appear to be fatal) of these studies. All data used in the manuscript have been made available for the purposes of reproducing the analysis.

 I was slightly confused by the description of the simulation study as a two-stage procedure in the critique of Roberts & Lowe. If I understand correctly, sets of simulated values for five quantities were drawn from Normal distributions centred around the estimates used by Roberts & Lowe, with standard deviations equal to these values multiplied by 0.2. Each time a new set of these five quantities was drawn, the values were used to calculate (predict) a value for embryo loss. This was done 100,000 times. However, the author speaks of 1,000 simulations, each containing 10,000 separate estimates. It is unclear what exactly varied within and between the 1000 simulations. If the data generating model was the same for all of these (ie: this was just done for computational reasons), then it would be helpful if the author could make this clear in the text.

 The author assumed that the simulated quantities were independent in the simulation – I confess to having no real intuition as to the implications of this assumption. However, I don’t believe this would affect the author’s conclusion.

 One minor typo; ‘this is far from being a robust pregnancy diagnosis and in different study [46]…’

 I believe that it would be appropriate to accept this manuscript without revision, although the author may wish to clarify the point about the first simulation described above.

I have read this submission. I believe that I have an appropriate level of expertise to confirm that it is of an acceptable scientific standard.

# Response

**Published:** 2017-04-30

**Authors:** Gavin Jarvis

**Affiliations:** University of Cambridge, UK

I would like to thank Dr Wilkinson for his helpful review. I hope the following clarify the points raised.

**Roberts & Lowe Simulation: **10,000 simulated records provided data to generate 1 each of the following parameters: mean, median, and 2.5 ^th^ & 97.5 ^th^ percentiles. Repeating this process generated slightly different values for these parameters owing to the specification of the random number generator. Hence, the “10,000 simulations step” was repeated to obtain 1,000 means, medians and percentile values. It is the means of these parameters that are reported. The 100,000 simulated records were generated separately, albeit using the same model structure, simply to produce data for the Figure. I have edited the text to clarify these points.

**Independence of random variables: **It is plausible to suppose that some of the random variables may be correlated, e.g., length of cycle and length of fertile period. However, they may not be, or the extent of any correlation may be weak. I do not believe that constructing a full variance-covariance matrix for the model would shed any further light on the precision of the estimates of embryo mortality. There are too many undefined and imprecise variables for the model to be quantitatively useful. A solution to this imprecision would be to obtain and use more robust estimates, rather than build a more complex statistical model.

**The minor typo** has been corrected. Thank you.

# Peer review: Referee response for version 1

**DOI:** 10.5256/f1000research.9616.r19546

**Published:** 2017-01-20

**Authors:** Philippa Saunders, Douglas A Gibson

**Affiliations:** 1 MRC Centre for Inflammation Reserach, University of Edinburgh, Edinburgh, UK; 2 MRC Centre for Inflammation Research, University of Edinburgh, Edinburgh, UK

The author has provided have provided a provocative and timely review of the evidence related to pregnancy success. The author has focused on the evidence as he sees it from four different categories of review published in the last few decades. 

 We think the title is appropriate and will attract attention and the abstract is generally well drafted but the final sentence ends rather abruptly. We suggest the author might wish to consider a more robust/informative ending to his abstract as this will be read alone in Pubmed.

 The following comments are provided in a spirit of trying to increase access to this article for a broader readership than might not be otherwise able to consider its contents, i.e. as currently written it seems largely to appeal to a people who might be interested in statistical analysis. Specifically we would like to see the author consider see how he might frame the evidence he provides alongside a timeline of the different stages of early pregnancy – this would mean individuals who are not well versed in reproductive function would be able to understand the arguments he is providing.

 We are pleased to see this article being written. We think it is timely, thought-provoking and this is an excellent moment in which to consider in realistic terms the kind of evidence that is constantly requoted in the debate about how fertile the human species is. Currently this topic is dominated by data from studies on women who are sub/infertile receiving medical support to achieve a pregnancy.

**Specific points**

  

1. Who is the audience for this paper and does the introduction set the scene in such a way that the reader will be both interested and motivated to read the remaining part of the paper, which I would like to see them do? I think as written the Introduction may not achieve this objective. For example the first sentence starts with some glib comments about it being ‘widely accepted’ that under natural circumstances human embryo mortality is high, and then there is an extensive section quoting number of popularist articles and websites – why have this up front? It seemed to undermine the erudite arguments of the rest of the paper.
2. The second paragraph with some modification would make a sufficient introduction. The aim of the review as stated in the discussion ‘How many fertilized human embryos..?’ should also be frontloaded at some point here. Clearly embryo mortality is of interest to both reproductive biologists and fertility doctors but why not also mention couples trying to conceive?
3. Reading the introduction we were struck by the pressing need for ’key terms’ box – the kind of thing you see in Nature papers – where there is a definition of each of the terms used, e.g. Fecundability, embryo, HCG, etc.  If this paper is going to be read by individuals who are not fertility experts or experts in reproductive biology but people interested in ethics or chance or statistics, I think they will be very confused by the different terms that are used.
4. What is not clear from the paper is the chronology of the observations/data being discussed. It is common for people (even those familiar with the field but who work on animal models) to be very confused by the timings in women. For example – the day on which fertilisation takes place versus the last menstrual period, e.g. fertilisation versus gestation versus the first day (depending on when you count from) on which you might reasonably expect to detect HCG in the urine. We would argue there needs to be a figure defining when each of these happens in terms of days in a woman's reproductive span. This could also help clarify the points in the process that the probabilities of π _FERT,_ π _CLIN _etc can apply to.
5. The second piece of information where we think it would be very helpful is under the section called 'What the data say' where the terms such as ‘old’ are added and there are no dates or refs provided. What do they mean by 'old' - pre 1960, pre 1950, pre 1940?
6. Because the author has used numbered references, there is also no sense of the relationship of one study to another in terms of dates i.e. how they chronologically relate to each other. Some minor reworking in which the author says, for instance, "the work of Hertig and Rogg in the 1950’s" would be helpful.
7. The author is also slightly confusing when talking about the pregnancy study (ref 42) not giving the names of the authors nor the date on which it was published in the section on page 4, and then in the reference, for instance in Fig 2, they talk about the pregnancy study ref 42 but in the figure it is shown as French and Bierman 1962. This is the kind of things that make it difficult to get a sense of the chronology of observations and how people have built on each other's observations in order to support subsequent studies, and this after all is one of the most crucial points of this paper.
8. On page 6 we finally get to some discussion about modern pregnancy tests. It is not until some pages after that we know whether they are in blood or urine. Mid cycle elevation of HCG - this is not defined in terms of days (cf comments above). For information the fact that these assays were likely to be urine-based assays is not mentioned until page 7.
9. We think many aspects of this paper are extremely well argued, very much so the data provided. The very great detailed analysis in Table 3 and also in other parts of page 7, and some very good points are made about the over-emphasis on using data from patient groups where infertility is probably one of the reasons for presentation that may have caused a less robust data set.
10. The author makes a valid argument about potential subfertility within the Hertig cohort but this is not balanced. Equally, these women were selected for proven fecunditity and this factor affects interpretation of this cohort as much as the other.
11. On page 10 the discussion starts with a key question how many fertilised human embryos die. It is slightly frustrating that this was not put up front as the question being addressed in this paper.  Maybe the author might like to consider setting out aims more clearly.
12. Again, in the discussion, many of the arguments being made would have been greatly enhanced by telling us the dates on which some of these studies were conducted. When looking at the reference list I see many of them were in the '80s and early '90s.
13. We wonder if the first paragraph on page 12 might reasonably be eliminated - it feels repetitive compared to other parts of the paper. I think the discussion of the studies by Macklon review ref 20 is extremely insightful and useful. However we draw the author's attention to a more recent study by Macklon and Brosens which we believe puts forward some interesting arguments that might reasonably be discussed in his study about how the endometrium in which the embryos are set to implant might be acting as a ‘sensor’ of embryo quality. This is in Biology Reproduction 2014, vol 91. There is also a complementary paper in Sci Rep, vol 6, Brosens *et al.* 2014.
14. The conclusion of the discussion seems more like a continuation of the critique of the final few paragraphs. It would be desirable to provide a concluding paragraph which holistically draws together the content of the review. Again the heavy use of quoting references as appears in the introduction masks the opportunity for the author to provide his own conclusions.

 

 In summary we welcome this review which we think makes many erudite comments on a difficult field.

We have read this submission. We believe that we have an appropriate level of expertise to confirm that it is of an acceptable scientific standard, however we have significant reservations, as outlined above.

# Response

**Published:** 2017-04-30

**Authors:** Gavin Jarvis

**Affiliations:** University of Cambridge, UK

I would like to thank Professor Saunders and Dr Gibson for their helpful review. I have tried to address each point as detailed below.

The abstract has been re-drafted to provide a clearer and more robust conclusion. I have also brought the “How many…” question posed in the discussion to the beginning of the Abstract and into the Introduction as suggested.

I hope that the changes made will make the article more accessible to a wider readership.

**Responses to specific points**

1. The article is intended primarily for a scientific audience, although I agree that it may have wider appeal and I would be pleased for it to be read as such. The references to claims in the press and popular media are important (see later); however, I agree that as an opening this may be a distraction. I have therefore moved this section to the discussion. The list of scientific citations that make claims about embryo mortality is essential since it substantiates my claim that high embryonic mortality is widely reported. Additionally, it illustrates the large variance in these estimates.
2. The introduction has been re-organised along the lines suggested. The second paragraph has become the start and the quantitative claims incorporated into it. The importance of embryo mortality to women trying to conceive is clear, and was already present in Version 1. It remains in Version 2 and has also been emphasised in the discussion. The “How many…” question has been frontloaded as suggested, and is now also in the abstract.
3. A glossary of key terms box has been provided.
4. A new figure provides a timeline for non-fecund and fecund cycles and key biological events.       
5. Most of the pre-1960 references are found in databases, e.g., PubMed; however, search terms appear less comprehensive as articles get older. Hertig (1959) (Ref. 90, PMID: 13613882) has no reference to pregnancy loss in its MeSH terms. Neither Rock & Hertig (1942) (Ref. 92) nor Opitz (2002) (Ref. 29) is indexed in PubMed. A PubMed search on {"early pregnancy loss"[All Fields]} generates 831 hits (25 ^th^ April 2017) of which the earliest is 1971.  However, I agree that the description in Version 1 is vague. I have removed the reference to “old”.
6. I have edited the text in several places to include names and dates. I hope this will assist readers in following the chronology. The hCG studies are already arranged by date in Table 3 and Figure 3.
7. Reference numbers have been added to the legend within the Figure, and names and dates included in the legend text to increase clarity.
8. All of the 13 studies measured hCG in urine except two. These two employed serum samples and have been identified in the text. The term “mid-cycle” has been removed and the meaning clarified using the new Figure 1.
9. Thank you for these comments.
10. I agree with the point made. In this section the first point I make about the 210 recruited women is that they were of “proven fertility”. The same point is made again in the commentary on Hertig’s data. I have also edited the text in response to Reviewer 3 and hope that the final result is appropriately balanced.
11. The question (slightly modified) has been included in the Abstract and Introduction. I hope that this helps to clarify and reinforce the purpose of the article.
12. Some dates have been incorporated to enhance chronological clarity (see point 6).
13. The first paragraph on page 12 addresses the importance of biological variance. It does not go into detail but stresses that point estimates of risk do not provide the whole picture when considering either populations or individual cases. As I have put it, a neglect of variance fosters a misleading appreciation of reality. I would prefer to retain this paragraph, in the hope that it will encourage readers to consider the importance and implications of numerical diversity when interpreting data.The arguments proposed by Macklon and Brosens relating to endometrial receptivity are indeed interesting. However, they propose mechanistic explanations for implantation failure and do not directly address the issue of how frequently such events occur. Nevertheless, their inclusion is contextually valuable, and I have made some comments on their studies.
14. The final paragraph has been edited. The quotations are useful to make it clear that I am not alone in drawing attention to the limitations of the available data. I have endeavoured to summarise the broad purpose and value of this work.
